# Lineage dynamics of the endosymbiotic cell type in the soft coral *Xenia*

**DOI:** 10.1038/s41586-020-2385-7

**Published:** 2020-06-17

**Authors:** Minjie Hu, Xiaobin Zheng, Chen-Ming Fan, Yixian Zheng

**Affiliations:** Department of Embryology, Carnegie Institution for Science, Baltimore, MD USA

**Keywords:** Cell biology, Transcriptomics

## Abstract

Many corals harbour symbiotic dinoflagellate algae. The algae live inside coral cells in a specialized membrane compartment known as the symbiosome, which shares the photosynthetically fixed carbon with coral host cells while host cells provide inorganic carbon to the algae for photosynthesis^1^. This endosymbiosis—which is critical for the maintenance of coral reef ecosystems—is increasingly threatened by environmental stressors that lead to coral bleaching (that is, the disruption of endosymbiosis), which in turn leads to coral death and the degradation of marine ecosystems^2^. The molecular pathways that orchestrate the recognition, uptake and maintenance of algae in coral cells remain poorly understood. Here we report the chromosome-level genome assembly of a *Xenia* species of fast-growing soft coral^3^, and use this species as a model to investigate coral–alga endosymbiosis. Single-cell RNA sequencing identified 16 cell clusters, including gastrodermal cells and cnidocytes, in *Xenia* sp. We identified the endosymbiotic cell type, which expresses a distinct set of genes that are implicated in the recognition, phagocytosis and/or endocytosis, and maintenance of algae, as well as in the immune modulation of host coral cells. By coupling *Xenia* sp. regeneration and single-cell RNA sequencing, we observed a dynamic lineage progression of the endosymbiotic cells. The conserved genes associated with endosymbiosis that are reported here may help to reveal common principles by which different corals take up or lose their endosymbionts.

## Abstract (web summary)

Single-cell RNA sequencing identifies the pattern of gene expression during lineage progression in endosymbiotic cells of the fast-growing soft coral *Xenia*, revealing principles that underlie uptake and maintenance of endosymbionts by this coral.

## Main

Many corals take up dinoflagellate algae of the Symbiodiniaceae family into their gastrodermis through feeding. Some cells in the gastrodermis, which lines the digestive tract, may have the ability to recognize particular types of algae. Through phagocytosis and by modulating host immune responses, the matching algal type is enclosed by endomembranes to form symbiosomes inside coral cells^1^. The symbiosome membrane is believed to contain transporters that mediate nutrient exchange between the algae and host cells^4^. Comparative transcriptome analyses on whole organisms using different cnidarian species before and after algae colonization or bleaching have identified genes, the up- or downregulation of which could contribute to endosymbiosis^5–7^. Comparative genomic and transcriptomic information in endosymbiotic and non-symbiotic cnidarian species has also been used to search for genes that may have evolved to mediate the recognition or endocytosis of Symbiodiniaceae^6–9^. However, these approaches do not differentiate whether the altered genes are expressed in the host endosymbiotic cells or other cell types without additional criteria. Protein inhibition or activation has also been used to suggest that host proteins containing C-type lectin domains, scavenger receptor domains or thrombospondin type 1 repeats are involved in uptake of algae and immunosuppression^10–12^. The broad expression and function of these proteins, coupled with potential off-target effects of inhibitors, greatly limit data interpretation. Therefore, a systematic description of genes and pathways that are selectively expressed in the host endosymbiotic cells is much needed to begin to understand the potential regulatory mechanisms that underlie the entry, establishment and—possibly—the expulsion of Symbiodiniaceae.

## Genome and single-cell transcriptome

**Fig. 1:**
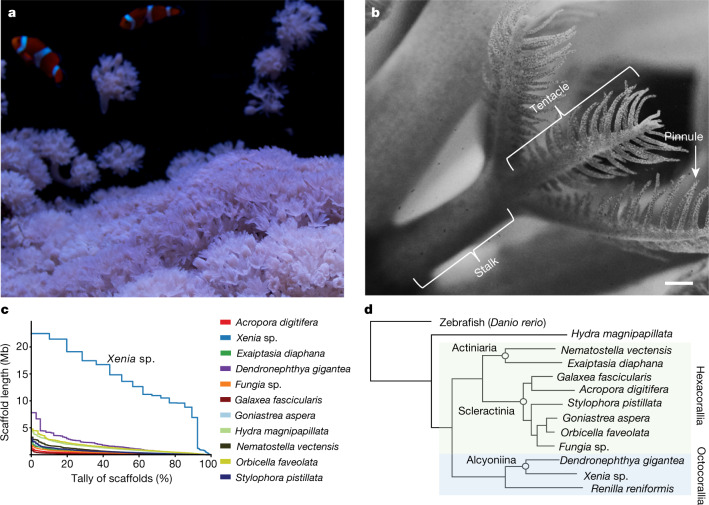
High-quality genome assembly for *Xenia* sp. **a**, *Xenia* sp. grown in the laboratory aquarium. **b**, An enlarged view of a *Xenia* sp. polyp with its main substructures indicated. Scale bar, 1 mm. **c**, Comparisons of the assembled scaffold lengths (*y* axis) and tallies (*x* axis) of 11 sequenced cnidarians, including *Xenia* sp. **d**, Evolutionary comparisons of *Xenia* sp. with other cnidarians, as indicated. Zebrafish and *Hydra* were used as outgroups. The phylogenetic branch points were assigned with 100% confidence. Source data

**Extended Data Fig. 1:**
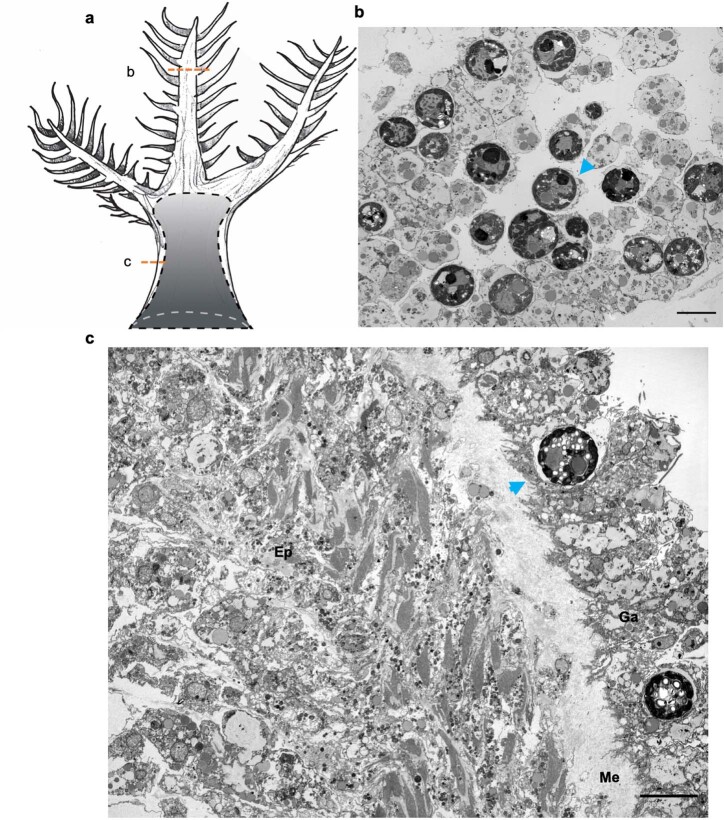
Electron microscopy analysis of *Xenia* sp. **a**, Illustration of a *Xenia* polyp. The orange dashed lines indicate where the electron microscopy images were taken, shown in **b** and **c**. **b**, **c**, Electron microscopy images. Ep, epidermis; Ga, gastrodermis; Me, mesoglea. Blue arrowheads, alga-containing *Xenia* cells. Five independent polyps from two independent experiments were used for electron microscopy. Scale bars, 10 μm.

**Extended Data Fig. 2:**
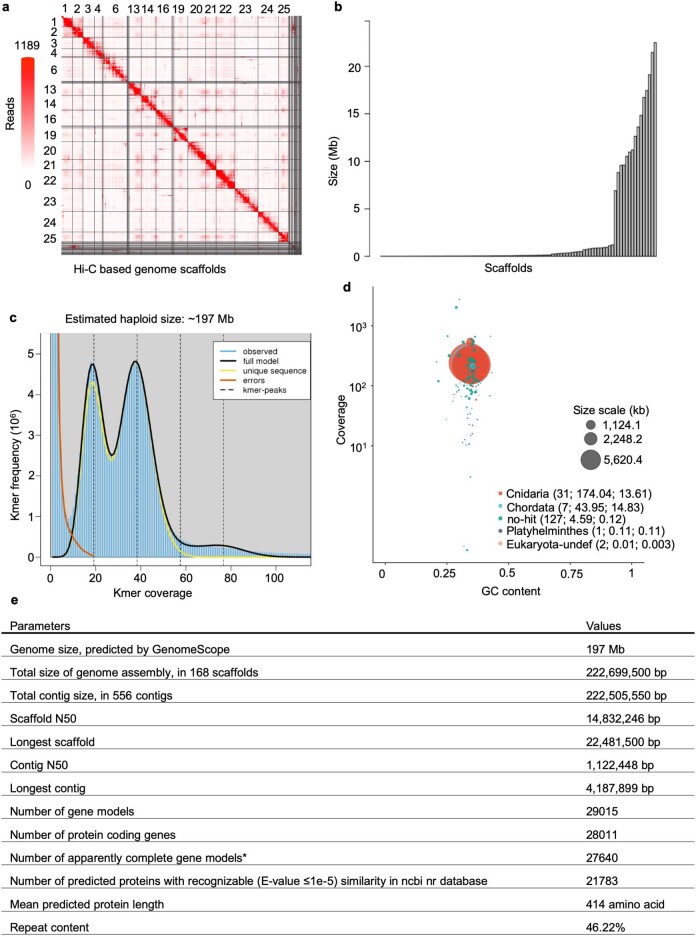
Additional genome assembly data. **a**, Hi-C-based *Xenia* sp. genome assembly. The scaffolds are separated by grids demarcated by black lines. The numbers for the 15 longest scaffolds out of the total 168 scaffolds are shown. **b**, Size distribution of *Xenia* sp. genome scaffolds. Each bar on the *x* axis represents a scaffold. **c**, *Xenia* sp. genome is predicted to be diploid, as expected, with a haploid genome size of about 197 Mb, on the basis of GenomeScope analysis of Illumina short reads. **d**, Contamination analysis by BlobTools revealed a similar GC content and genomic coverage across most scaffolds. Each coloured circle in the graph represents a scaffold. Larger circles have longer scaffold lengths; the three grey circles provide the length scale used in the plot. The colour codes represent the closest species group that has the highest sequence similarities to the *Xenia* sp. scaffolds (the first number in each set of parentheses shows the *Xenia* scaffold number followed by the combined length of the scaffolds and scaffold N50 value (minimum contig length needed to cover 50% of the combined scaffold length) in Mb). **e**, A summary of *Xenia* sp. genome assembly and gene annotation. *Genes encoding protein sequences with apparent in frame start and stop codons.

We chose to study a *Xenia* sp. of pulsing soft coral (Fig. 1a, b, Extended Data Fig. 1, Supplementary Video 1) that grows rapidly in a laboratory aquarium. Using Illumina short-read and Nanopore long-read sequencing (Extended Data Table 1), we assembled the *Xenia* genome into 556 high-quality contigs. Applying chromosome conformation capture (Hi-C)^13,14^, we further assembled these contigs into 168 scaffolds; the longest 15 of these scaffolds contain 92.5% of the assembled genome of 222,699,500 bp, consistent with the GenomeScope estimation (Extended Data Fig. 2). To our knowledge, the *Xenia* genome has by far the longest scaffold length, and thus the most contiguous assembly, of the published cnidarian genomes (Fig. 1c). Annotation using several bulk RNA-sequencing (RNA-seq) datasets showed that *Xenia* sp. has 29,015 genes, similar to other cnidarians (Extended Data Tables 2, 3). Consistent with previous phylogenetic analyses^15^, the octocorallians, *Xenia* sp., *Dendronephthya gigantea* and *Renilla reniformis* are grouped as a clade that is sister to the hexacorallian clade (which contains sea anemones and scleractinian corals), as they are all anthozoans (Fig. 1d).

**Fig. 2:**
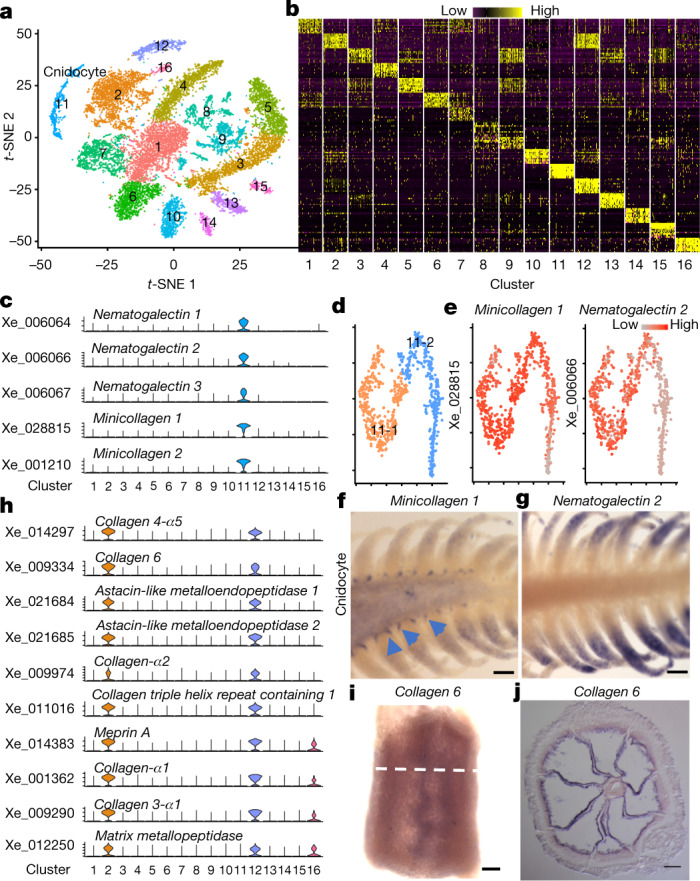
scRNA-seq transcriptomes suggest that there are 16 cell types in *Xenia* sp. **a**, Transcriptomes of 19,134 individual *Xenia* sp. cells obtained by scRNA-seq were grouped into 16 clusters (colour-coded) and presented in *t*-SNE space. Each coloured dot represents one cell. **b**, Gene-expression heat map (scale at the top) for the top 10 gene markers that define each cluster. Each column represents one cell cluster, and each row represents one gene. Forty cells were randomly selected from each of the 16 cell clusters for plotting. **c**, Expression profiles of the indicated cluster-11 *Xenia* (Xe) marker genes out of all cell clusters. **d**, Cluster-11 cells are subdivided into two populations (11-1, 423 cells; 11-2, 374 cells, colour-coded) and displayed in a *t*-SNE space. Each coloured dot represents a cell. **e**, Expression levels (scale to the top right) of two cluster-11 markers, *Minicollagen 1* and *Nematogalectin 2*, are shown in a *t*-SNE plot. *n* = 797 cells. **f**, **g**, Whole-mount RNA ISH of *Minicollagen 1* (**f**) and *Nematogalectin 2* (**g**), showing their expression in tentacles. Arrows indicate the expression of *Minicollagen 1* at the base of pinnules. **h**, Expression profiles of marker genes enriched in clusters 2, 12 and 16 out of all 16 clusters. **i**, **j**, RNA ISH of *Collagen 6*. Whole-mount view of the stalk in **i** and cross-section image in **j**. The white dashed line in **i** indicates the cross-section level in **j**. More than 12 polyps from 4 independent experiments were used for each probe. Scale bars, 100 μm (**f**, **g**, **j**), 150 μm (**i**). Cell numbers for clusters 1–16 are 2,794; 2,704; 2,073; 1,679; 1,511; 1,374; 1,248; 1,069; 986; 923; 797; 649; 575; 321; 246; and 185, respectively (**a**, **c**, **h**).

**Extended Data Fig. 3:**
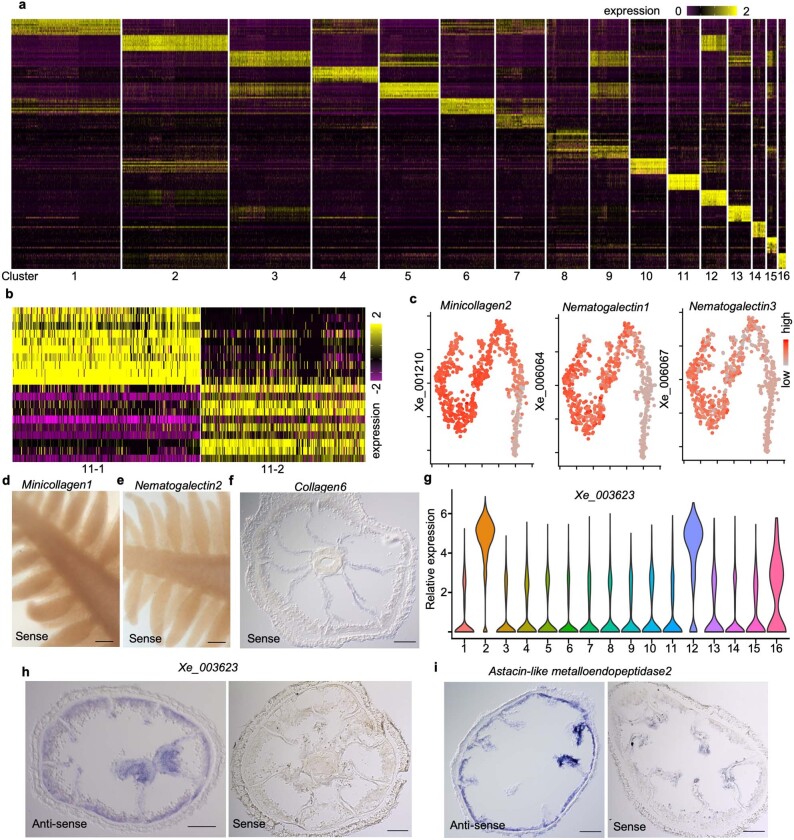
Additional scRNA-seq analyses. **a**, Heat map showing differential gene expression patterns of all cells in the 16 assigned cell clusters (indicated at the bottom). Each column is one cell cluster, and each row represents one gene. **b**, Heat map showing differential gene expression patterns of two subclusters in cluster 11 (11-1 and 11-2). **c**, Expression levels (as in the coloured expression scale) of 3 cluster-11 markers, *Minicollagen 2*, *Nematogalectin 1* and *Nematogalectin 3*, are shown in the *t*-SNE plots. *n* = 797 cells. **d**–**f**, RNA ISH control with sense probe for *Minicollagen 1* (**d**), *Nematogalectin 2* (**e**) and *Collagen 6* (**f**). **g**, Expression levels of *Xe_003623*, a non-conserved and uncharacterized cluster-2 and -12 marker gene, in each of the 16 cell types defined by scRNA-seq. Violin plot (Methods) show the distribution of gene expression in each of the 16 clusters. Cell numbers in cell clusters 1–16 were 2,794; 2,704; 2,073; 1,679; 1,511; 1,374; 1,248; 1,069; 986; 923; 797; 649; 575; 321; 246; and 185, respectively. **h**, **i**, RNA ISH of *Xe_003623* (**h**) and *Astacin-like metalloendopeptidase 2* (**i**) using anti-sense and sense probes. In **d**–**f**, **h**, **i**, more than 12 polyps from 3 independent experiments were used for each probe. Scale bars, 100 μm.

We next performed single-cell RNA-seq (scRNA-seq)^16^ of whole polyps, stalks or tentacles using version 2 and version 3 chemistry of the 10x Genomics platform (Supplementary Table 1, Methods). Using *t*-distributed stochastic neighbour embedding (*t*-SNE)^17^, we grouped the high-quality single-cell transcriptomes, covering 23,939 genes, into 16 cell clusters with distinct gene-expression patterns (Fig. 2a, b, Extended Data Fig. 3a, Supplementary Table 2). For validation, we looked for two previously characterized cnidarian cells: the cnidocytes, which are used for prey capture and/or defence, and gastrodermal cells. The cells of cluster 11 express minicollagen and nematogalectin genes, which are markers of cnidocytes^18–20^ (Fig. 2c). Further analysis revealed that cluster 11 contained two subclusters (Fig. 2d, Extended Data Fig. 3b). Minicollagen genes are expressed in both subclusters, whereas nematogalectin genes are preferentially expressed in one (Fig. 2e, Extended Data Fig. 3c). RNA in situ hybridization (ISH) confirmed the expression of a nemetogalectin gene to be more spatially restricted than that of *Minicollagen 1* in *Xenia* pinnules (Fig. 2f, g, Extended Data Fig. 3d, e). Clusters 2, 12 and 16 express genes that encode collagens and proteases (Fig. 2h) that are known to be enriched in gastrodermis of *Nematostella*^18^. RNA ISH for *Collagen 6*, *Astacin-like metalloendopeptidase 2* (both expressed by clusters 2 and 12) and the uncharacterized *Xe_003623* gene (expressed by clusters 2, 12 and 16) confirmed the high expression of these genes in the gastrodermis (Fig. 2h–j, Extended Data Fig. 3f–i). Thus, the clustering analyses and ISH identified cnidocytes and cells in the gastrodermis in *Xenia*.

## Endosymbiotic cell type in *Xenia* sp.

**Fig. 3:**
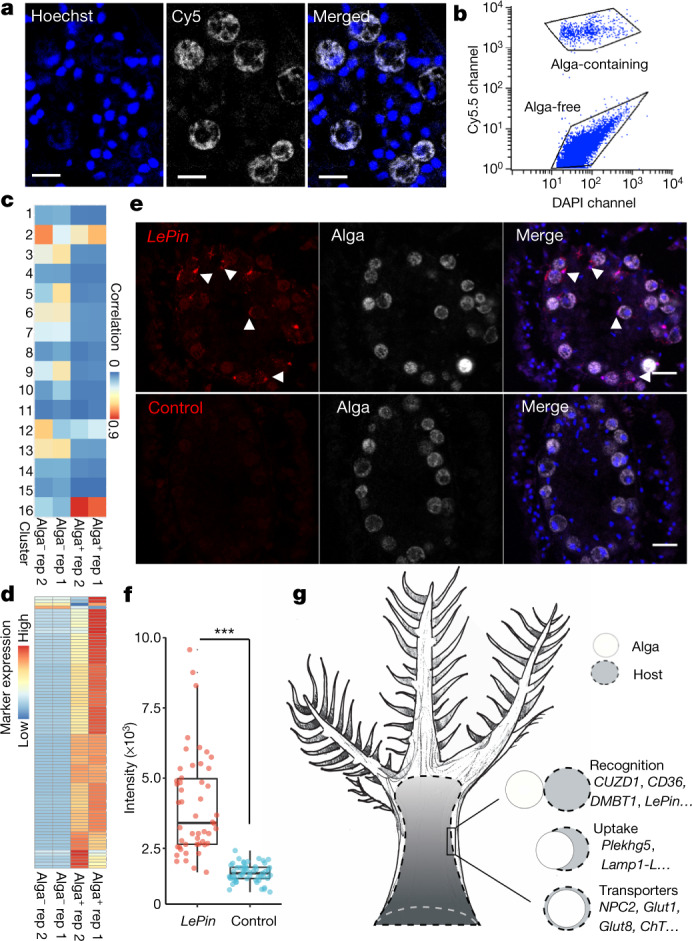
Identification of genes specifically expressed in *Xenia* sp. endosymbiotic cells. **a**, The endosymbiotic algae in *Xenia* display autofluorescence in the Cy5.5 far-red channel. A cross-section of *Xenia*, with the *Xenia* and algal nuclei stained by Hoechst (blue) and alga autofluorescence (white). **b**, A FACS profile of dissociated live *Xenia* cells using Cy5.5 and DAPI channels. Five biological replicates (**a**, **b**). **c**, Pearson correlation of gene expression between the scRNA-seq data of 16 cell clusters and the bulk RNA-seq data of 2 biological replicates of FACS-isolated alga-containing (alga^+^ rep 1 and alga^+^ rep 2) and alga-free (alga^−^ rep 1 and alga^−^ rep 2) cells. **d**, Heat map showing the expression levels of the 89 marker genes for cluster-16 cells in alga-containing and alga-free cells, isolated by FACS. **e**, Ultra-sensitive fluorescence RNA ISH by RNAscope probing for *LePin* (red) (top) and control (bottom). White arrows indicate the *LePin* signal. Hoechst staining of all nuclei is shown in blue. Scale bars, 20 μm. **f**, Quantification of *LePin* signals. The fluorescence signal surrounding each alga is quantified in random sections and plotted (a dot = a section) for *LePin* and controls. ***P = 2.84 × 10^−16^, two-sided *t*-test. Lines in the box denote the median; the upper and lower edges of the box represent the upper and lower quartiles, respectively. Nine polyps from three independent experiments were used for each probe (**e**, **f**). **g**, Illustration of steps through which *Xenia* endosymbiotic cells may recognize and take up algae to establish endosymbiosis, with some candidate genes shown at each step. Source data

**Extended Data Fig. 4:**
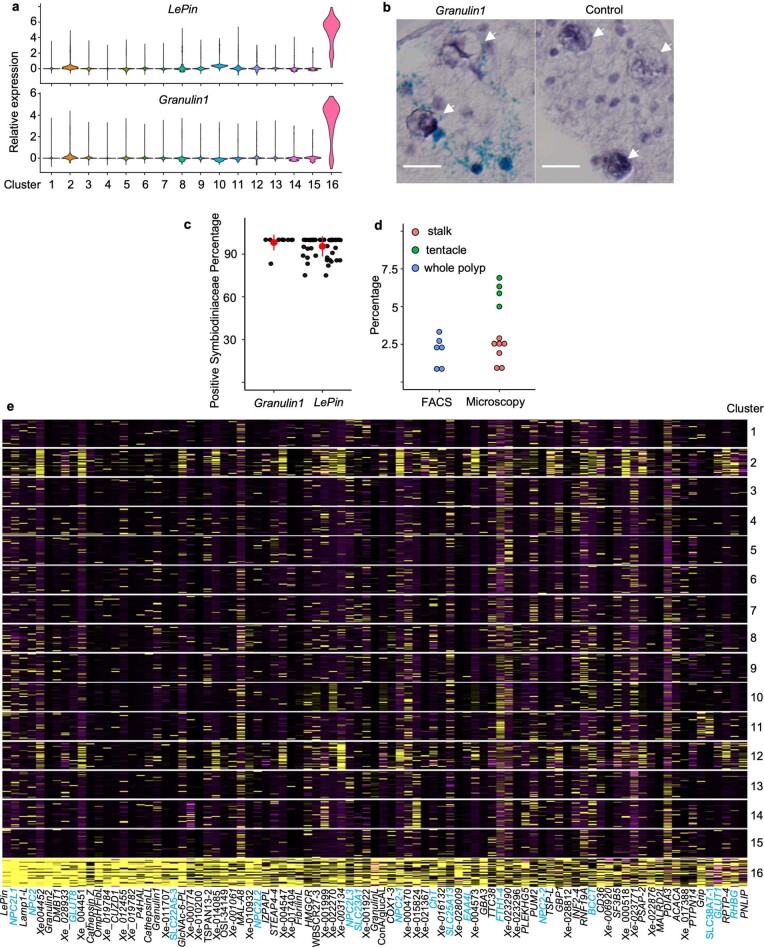
Additional analyses for endosymbiotic cells. **a**, Violin plots of the expression profiles of *LePin* and *Granulin 1* in the 16 clusters defined by scRNA-seq. Violin plots show the distribution of gene expression in each of the 16 clusters. Cell numbers in cell clusters 1–16 are 2,794; 2,704; 2,073; 1,679; 1,511; 1,374; 1,248; 1,069; 986; 923; 797; 649; 575; 321; 246 and 185, respectively. **b**, Ultra-sensitive chromogenic RNA ISH by RNAscope probing for *Granulin 1* (left) and control (right). Positive signals are blue. Nuclei were counterstained as purple with haematoxylin. White arrows indicate algae of the Symbiodiniaceae. Six polyps from three independent experiments were used for each probe. Scale bars, 10 μm. **c**, Percentage of alga-containing cells with positive *Granulin 1* or *LePin* signal. Each black dot stands for one section. Red dots and lines stand for mean and s.d., respectively. Six polyps from three independent experiments were used for one gene or control. **d**, Percentage of alga-containing cells measured by FACS and microscopy. For FACS, each dot stands for an individual polyp. Three independent experiments were performed with each experiment, using two polyps. For microscopy, each dot stands for a section analysed in three polyps. **e**, Heat map showing the enrichment levels of the 89 marker genes in cluster 16 among all 16 cell clusters. Transporters are highlighted in blue Source data.

To identify the cells that perform endosymbiosis, we took advantage of the autofluorescence of the member of the Symbiodiniaceae (*Durusdinium*) in our *Xenia* sp. (Methods). Using fluorescence-activated cell sorting (FACS), we separated alga-containing and alga-free *Xenia* cells (Fig. 3a, b) and performed bulk RNA-seq (Supplementary Table 3). By comparing these bulk transcriptomes with genes expressed in each cluster, we found that cells of cluster 16 exhibited the highest overall similarity to the alga-containing cells and most of the marker genes for cluster 16 (Supplementary Table 4) have a higher level of expression in alga-containing *Xenia* cells than that in alga-free *Xenia* cells (Fig. 3c, d, Supplementary Table 5). RNAscope ISH for two of the cluster-16 marker genes—one of which encodes a protein with lectin and kazal protease inhibitor domains (abbreviated LePin, encoded by a gene that we name *LePin*), and the other of which encodes Granulin 1—showed that these genes were expressed in alga-containing gastrodermal cells (Fig. 3e, f, Extended Data Fig. 4a, b). Additionally, on average 95% and 98% of alga-containing *Xenia* cells were positive for expression of *LePin* and *Granulin 1*, respectively (Extended Data Fig. 4c). On the basis of microscopy of cryopreserved tissue sections or FACS analyses, we estimated that on average 2–6% of *Xenia* cells contained algae and that tentacles have a higher percentage of alga-containing cells than do stalks (Extended Data Fig. 4d, Methods). This is consistent with the cluster-16 endosymbiotic cells being identified by scRNA-seq as a small fraction (382 cells, 1.4% of the total). Of the three gastrodermal cell clusters, cluster-16 cells therefore have a high likelihood of being a major cell type involved in endosymbiosis.

**Extended Data Fig. 5:**
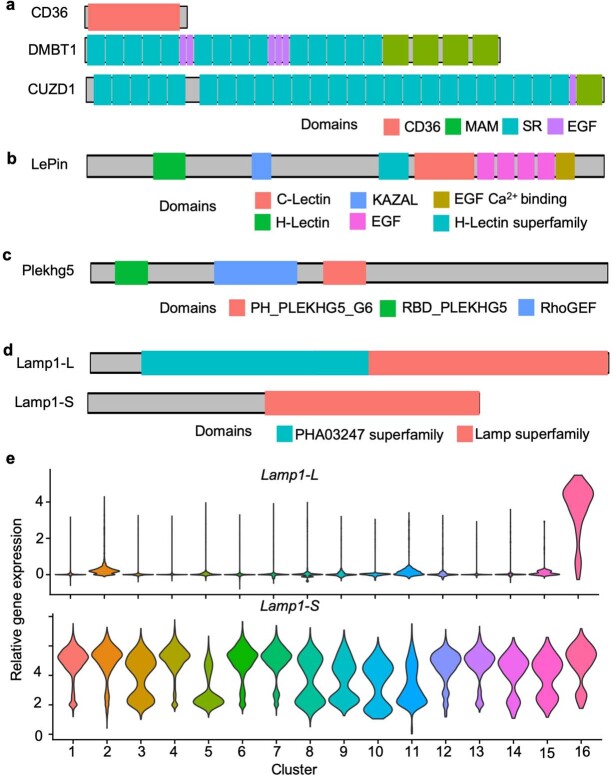
Selected endosymbiotic markers with known domains. **a**, The scavenger receptors (SR) CD36, DMBT1 and CUZD1. **b**, LePin. **c**, Plekhg5. **d**, Lamp1-L and Lamp1-S. **e**, Violin plots of expression profiles of *Lamp1-L* and *Lamp1-S* in 16 clusters defined by scRNA-seq. Violin plots show the gene-expression distribution in each cluster. Cell numbers in cell clusters 1–16 are 2,794; 2,704; 2,073; 1,679; 1,511; 1,374; 1,248; 1,069; 986; 923; 797; 649; 575; 321; 246 and 185, respectively.

Among the top 89 marker genes enriched in the cluster-16 endosymbiotic cells, 67 encode proteins with domains of known or predicted functions, including receptors, extracellular matrix proteins, immune response proteins, phagocytosis and/or endocytosis proteins, or nutrient transporters (Extended Data Fig. 4e, Supplementary Table 4). Three proteins—encoded by *CD36*, *DMTB1* and *CUZD1*—contain CD36 or scavenger receptor domains that are known to recognize a wide range of microbial surface ligands and mediate their phagocytosis, and that also modulate the innate immune response of the host^11,21,22^ (Fig. 3g, Extended Data Fig. 5a). CUZD1 is the least understood, and is similar to DMBT1 in domain organization. DMBT1 functions in pattern recognition of microorganisms. In mammals, it is expressed on the surface of the gastrointestinal tract, where it recognizes polysulfated and polyphosphorylated ligands on microorganisms, represses the inflammatory response and regulates the differentiation of gastrointestinal cells^23^. *LePin* and *Granulin 1*, which we used for ISH, have homologues in *Exaiptasia*, as well as stony and soft corals. Because LePin has an N-terminal signal peptide followed by multiple domains (including H- and C-type lectins and a Kazal-type serine protease inhibitor) (Extended Data Fig. 5b), it may confer selectivity for the Symbiodiniaceae. On the basis of previous studies of granulins in mammals^24^, *Granulin 1* may modulate the immune response in *Xenia* endosymbiotic cells.

Phagocytosis of the Symbiodiniaceae by gastrodermal cells (which are of a similar size to these algal cells) requires substantial expansion of the host cell, but the genes that regulate this size expansion are unknown. Among the endosymbitoic marker genes that we found, *Plekhg5* encodes a highly conserved RhoGEF (Fig. 3g, Extended Data Fig. 5c). In *Xenopus*, Plekhg5 localizes to the apical membrane of epithelial cells and recruits actomyosin to induce cell elongation and apical constriction^25^. Thus, *Plekhg5* is a prime candidate for regulating the extension of the apical membrane to engulf algae of the Symbiodiniaceae during the early stages of phagocytosis in *Xenia*. Upon phagocytosis, algae of the Symbiodiniaceae are enclosed by the host membrane to form symbiosomes^26^. Although the symbiosome is acidified similarly to lysosomes^27^, the genes that are involved in the formation of the symbiosome remain unclear. *Xenia* sp. has two genes that encode lysosome-associated membrane glycoproteins, which are more similar to the previously characterized LAMP1 than to LAMP2^28^. In *Xenia*, *Lamp1-L* encodes a larger protein and is an endosymbiotic marker gene, whereas *Lamp1-S* encodes a smaller protein and is expressed across all cell clusters (Extended Data Fig. 5d, e). Because lysosome-associated membrane glycoproteins are known to regulate phagocytosis, endocytosis, lipid transport and autophagy^28^, Lamp1-L may regulate symbiosome formation and/or function (Fig. 3g). Several endosymbiotic marker genes encode enzymes that may promote the establishment of endosymbiosis or facilitate nutrient exchanges between alga and the host cell. For example, there are 17 genes that potentially participate in nutrient exchanges, as they encode transporters for sugar, amino acids, ammonium, water, cholesterol and choline (Fig. 3g, Extended Data Fig. 4e, Supplementary Table 4).

## Lineage dynamics of endosymbiotic cells

**Fig. 4:**
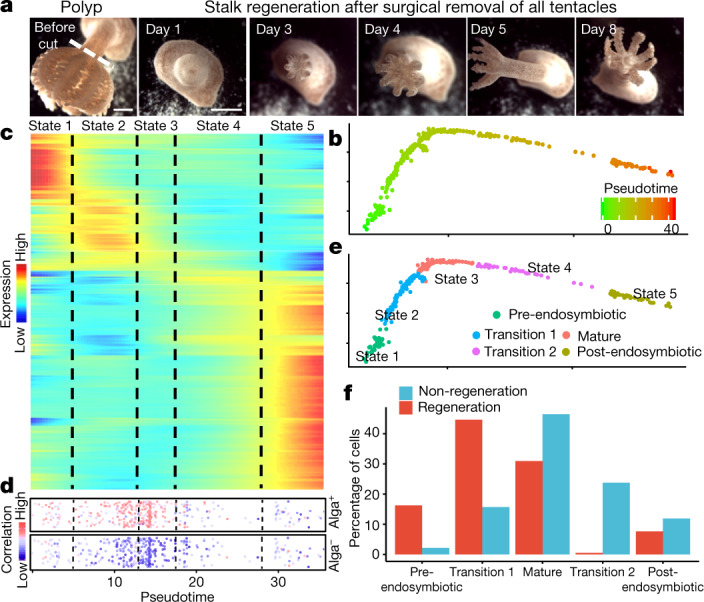
Dynamic lineage progression of endosymbiotic cells. **a**, An example of a *Xenia* sp. polyp (shown in the panel on the far left) is surgically cut at the white dashed line to remove all the tentacles. A surgically cut stalk is shown to regenerate in successive days as indicated. Five biological replicates. Scale bars, 1 mm. **b**, Pseudotime trajectory of all endosymbiotic cells (a dot represents a cell) identified in regeneration and non-regeneration scRNA-seq datasets. The pseudotime indicator is shown at the bottom right. **c**, Heat map for gene-expression levels along pseudotime. **d**, Correlation of the scRNA-seq transcriptome with the bulk RNA-seq transcriptome of alga-containing or alga-free *Xenia* cells, isolated by FACS. The endosymbiotic cells used to model gene expression along the pseudotime line are aligned with the heat map. Each cell represented by a dot is coloured according to the Pearson correlation of its transcriptome with the indicated bulk RNA-seq transcriptome. Five cell states (states 1–5, separated by dashed lines) are defined by differential gene expression together with the Pearson correlation. **e**, Five states defined by **c** and **d**. **f**, The percentage of cells in each state in regeneration and non-regeneration samples.

**Extended Data Fig. 6:**
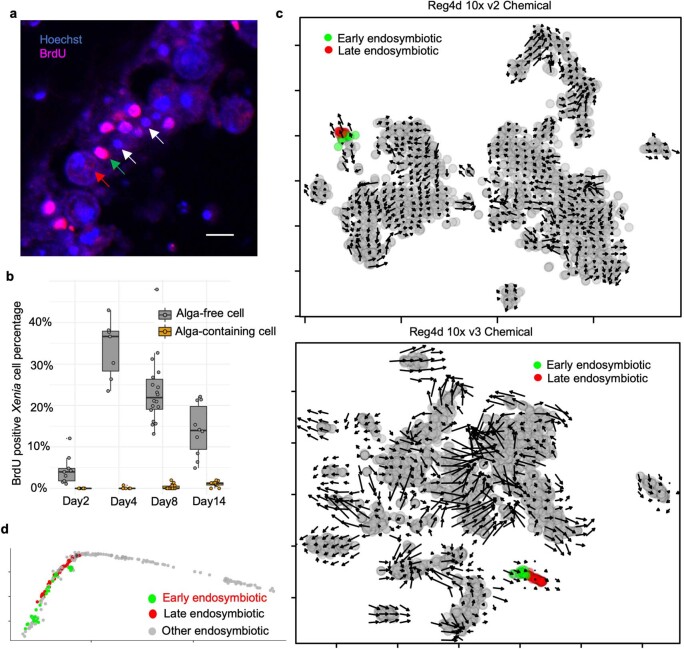
Additional analyses of endosymbiotic cell lineage. **a**, A representative image of BrdU labelling (pink), overlaid with Hoechst (blue DNA stain) in a cross-section of a regenerating *Xenia* sp. stalk. White, red and green arrows indicate BrdU-negative (BrdU^−^) *Xenia* nuclei, an alga and a BrdU-positive (BrdU^+^) *Xenia* nucleus juxtaposed to the alga, respectively. Three regenerating stalks were used in two independent experiments. **b**, Box plot. Percentages (*y* axis) of BrdU^+^ *Xenia* cells at the indicated regeneration time points (*x* axis). Each dot represents data from one section. Three regenerating stalks from two independent experiments were pooled and plotted for each time point. About 7 to 18 sections were used for each group. The alga-containing proliferated *Xenia* cells were estimated as those with BrdU^+^ nuclei juxtaposed to algae. The medians are indicated as lines in the box; the upper and lower edge of the boxes represent the upper and lower quartiles, respectively. **c**, Velocyto analysis of the scRNA-seq data from day-4 regenerating *Xenia* sp. stalks. Each dot represents a cell, and arrows indicate the directions of RNA velocity. The green and red endosymbiotic cell clusters were predicted as early and late-cell states, respectively, on the basis of the directions of the arrows. **d**, The distribution of early (green) and late (red) endosymbiotic cells predicted by velocyto in **c** on the pseudotime plot of all scRNA-seq data shows the start and the direction of progression of the endosymbiotic cell lineage. Source data

To better understand the temporal dynamics of cluster-16 cells, we developed a *Xenia* regeneration model. We surgically cut away all tentacles from *Xenia* polyps and found that the stalks regenerated all tentacles in several days when cultured in the seawater from our aquarium that houses stock corals (Fig. 4a). Individual tentacles also regenerated into full polyps, but required a longer time (data not shown). BrdU labelling showed that some proliferated (BrdU^+^) gastrodermal cells began to take up algae that were present either in the gastrodermis or in the seawater at day 4 of regeneration (Extended Data Fig. 6a, b). We performed scRNA-seq of the regenerating stalks and pooled the data with the scRNA-seq of non-regenerating samples (Methods).

We used Monocle 2 to perform pseudotemporal ordering of all of the endosymbiotic *Xenia* cells^29^ (Fig. 4b); Monocle 2 uses reversed graph embedding to construct a principal curve that passes through the middle of the cells in the *t*-SNE space. Because this trajectory analysis does not provide a direction of cell-state progression, we used velocyto^30^ to determine the directionality of lineage progression of all cells and focused on the endosymbiotic cells in the regenerating sample. Velocyto calculates RNA velocity by comparing the number of unspliced and spliced reads, which measures the expected change in gene expression in the near future—thereby providing the directionality of cell-state change. This enabled the identification of early and late stages of endosymbiotic cells (green and red, respectively, in Extended Data Fig. 6c). The cell trajectory showed that the early and late-stage cells are mapped to relatively early and late pseudotime, respectively (Extended Data Fig. 6d). Thus, the pseudotime represents actual lineage progression. Modelling of gene expression revealed substantial changes along pseudotime. Further hierarchical clustering showed distinct gene-expression patterns, which helped to define five putative endosymbiotic cell states (Fig. 4c, Supplementary Table 6).

To further explore the cell dynamics in these five states, we compared single-cell transcriptomes to transcriptomes from the bulk RNA-seq of alga-containing or alga-free cells isolated by FACS, and plotted the expression correlation along pseudotime. State-3 cells showed the strongest correlation with the alga-containing cells, followed by state 2 and then state 1; state-4 and state-5 cells showed the least correlation (Fig. 4d). This suggests that state 3 represents mature, alga-containing cells. State-1 and state-5 cells showed correlations with alga-free cells (Fig. 4d). Given that these five states are present in our identified endosymbiotic cell type with a linear pseudotime progression, we hypothesize that state-1 cells are pre-endosymbiotic progenitors that can transition through state 2 to become state-3 mature alga-containing cells, and that state-3 cells could further transit through state 4 into state-5 post-endosymbiotic cells (Fig. 4e). In support of this, we found that the regenerating samples have higher percentages of state-1 (pre-endosymbiotic) and state-2 (transition 1) cells, and that the non-regeneration sample has more state-3 mature, state-4 (transition 2) and state-5 (post-endosymbiotic) cells (Fig. 4f).

**Extended Data Fig. 7:**
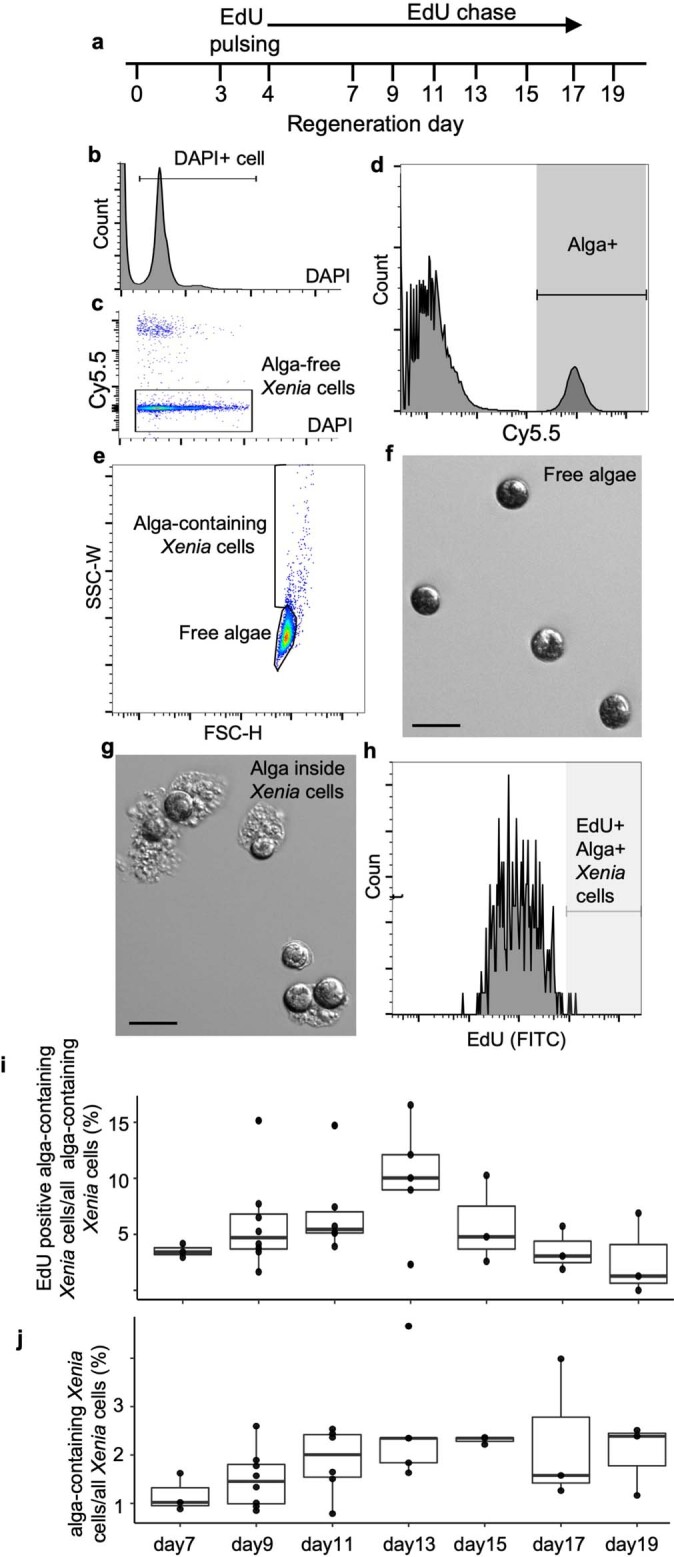
EdU pulse–chase analysis of endosymbiotic cells. **a**, Pulse–chase experiments. The regeneration stalk was labelled with EdU at regeneration day 3 and day 4. After washing out EdU, the samples were cultured, collected and analysed at the indicated days during chasing. **b**, Dissociated cells were processed by Click-iT to visualize EdU and stained with DAPI to label nuclei. Cells were sorting on the basis of DAPI. **c**, DAPI-positive cells were further sorted on the basis of Cy5.5 to estimate the number of the total alga-free *Xenia* cells. **d**, **e**, To estimate the number of alga-containing *Xenia* cells, free algae and alga-containing *Xenia* cells (alga^+^ population) were first separated from all the other *Xenia* cells on the basis of the Cy5.5 signal (**d**). The alga^+^ population was further separated into alga-containing *Xenia* cells and free algae based on the SSC and FSC signals (**e**). **f**, **g**, Microscopy confirmation of free algae (**f**) and alga-containing *Xenia* cells (**g**) sorted in **e**. Scale bars, 20 μm. In **c**–**h**, four independent experiments were carried out. **h**, The number of EdU-positive and alga-containing *Xenia* cells were further estimated on the basis of their strong EdU signal. **i**, Box plot of the percentage of EdU-positive and alga-containing *Xenia* cells among all alga-containing *Xenia* cells at the indicated days of chase. **j**, Box plot of the percentage of all alga-containing *Xenia* cells among all *Xenia* cells at the indicated days of chase. Each dot in **i**, **j** stands for one regenerating sample. Day 7, *n* = 3 polyps; day 9, *n* = 8 polyps; day 11, *n* = 6 polyps; day 13, *n* = 5 polyps; day 15, *n* = 3 polyps; day 17, *n* = 3 polyps; day 19, *n* = 3 polyps from 2 independent experiments were assayed. The medians are indicated as lines in the boxes; the upper and lower edges of the boxes represent the upper and lower quartiles, respectively. Source data

We further verified our hypothetical endosymbiotic cell states in the regeneration paradigm by pulse–chase experiments (Methods). After cutting, *Xenia* sp. stalks were pulsed with EdU at day 3 and day 4 of regeneration. EdU was washed out, corals were allowed to continue regenerating and samples were collected at days 7, 9, 11, 13, 15, 17 and 19 (Extended Data Fig. 7a). Using FACS (Extended Data Fig. 7b–h), we calculated the percentages of EdU^+^ alga-containing cells out of all alga-containing *Xenia* cells, and the percentages of all alga-containing *Xenia* cells out of all *Xenia* cells. We found an increase of EdU^+^ alga-containing *Xenia* cells up to regeneration day 13, which may account for the increase in uptake of algae during tentacle growth (as tentacles have more alga-containing cells than the stalk) (Extended Data Figs. 4d, 7i). Thereafter, the percentage of total alga-containing cells remained constant, but the percentage of EdU^+^ alga-containing cells gradually decreased (Extended Data Fig. 7i, j). Thus, these results support our hypothesis that the endosymbiotic cells progress from a progenitor state through an alga-uptake state and a mature alga-containing state, followed by loss of their algae.

Analysis of differentially expressed genes suggests the roles of each state in endosymbiotic cell lineage development and function. For example, the state-1 pre-endosymbiotic cells express *WNT7b* and *WNT11*, which may regulate progenitor-cell proliferation and differentiation through the Wnt signalling pathway^31,32^. Among 24 genes preferentially expressed in state 3, 13 are endosymbiotic markers that are expressed at higher levels in the FACS-isolated alga-containing *Xenia* cells than that in the alga-free cells. By contrast, none of the genes preferentially expressed in state 5 is an endosymbiotic marker. Instead, state-5 cells preferentially express several oxidative-stress-response genes (see Supplementary Table 6 for detailed descriptions). Because increased oxidative stress is observed upon cellular ageing and during coral bleaching^33–35^, state-5 cells may represent a natural ageing state of endosymbiotic cells that are no longer able to hold on to their algae. Additional molecular studies exploring the function of the differentially expressed genes in each state are needed to further validate our five-state hypothesis.

## Summary and outlook

Here we demonstrate the power of genomic and bioinformatic tools in studying coral biology. The *Xenia* sp. genome encodes essential components of RNA interference, such as Dicer and Ago, and DNA repair pathway proteins, which should enable the development of gene-manipulation tools to determine the mechanism of endosymbiosis. Although we focused on studying the endosymbiotic cell lineage, the regenerative processes for the other cell clusters can be similarly investigated in future analyses. Our studies suggest that *Xenia* endosymbiotic cells exist in five progressive states that are dynamic between homeostatic conditions and the regeneration process (Fig. 4f). It will be important to further understand the endosymbiotic lineage progression under different environmental stressors and to test whether efficient recovery from bleaching relies on state-1 pre-endosymbiotic cells. It is also feasible to test whether forced regeneration by fragmenting bleached corals can stimulate the expansion of state-1 pre-endosymbiotic cells and the restoration of endosymbiosis.

## Methods

No statistical methods were used to predetermine sample size. The experiments were not randomized and investigators were not blinded to allocation during experiments and outcome assessment other than the bioinformatic analyses.

### Coral aquarium

The coral aquarium is established in a tank (Reefer 450 system, Red Sea). The artificial seawater, made from Coral Pro Salt (Red Sea), was first incubated with live rocks for two months before introducing *Xenia* sp., other corals, fish, snails and hermit crabs. The aquarium is maintained at about 80 °F with about 25% change of seawater every 1–2 weeks. The light is provided by Hydra 26 HD LED (Aqua Illumination) with 60% power on during 10:00 to 19:00. The fish were fed with fish pellets (New Life Spectrum Marine Fish Formula) and Green Marine Algae (Ocean Nutrition).

The *Xenia* sp. was obtained from a local coral aquarium shop called CTE Aquatics. We performed taxonomy analysis by amplifying *ITS2* rDNA region of Symbiodiniaceae species with primers (SYM_VAR_5.8S2, GAATTGCAGAACTCCGTGAACC and SYM_VAR_REV, CGGGTTCWCTTGTYTGACTTCATGC)^36^. Sequence analysis showed that the *Xenia* sp. in our aquarium contains multiple Symbiodiniaceae species, of the genus *Durusdinium*. In all of our experiments, samples of polyps or colonies were randomly selected from the aquarium. We selected polyps that appeared fully grown in size, and colonies that were easy to break off from their attachment sites. We will share our live *Xenia* sp. with any researchers upon request. We have also deposited some frozen and fixed coral colonies, along with genomic DNA and total RNA, at the Smithsonian National Museum of Natural History (catalogue no., USNM 1613385).

### Genomic DNA isolation from *Xenia* sp.

To enable Nanopore DNA sequencing, we modified a protocol^37^ that allowed the isolation of long DNA fragments. For each DNA preparation, one or two *Xenia* sp. colonies containing about 30 polyps were collected from the aquatic tank and washed 3 times for 5 min each with Ca^2+^- and Mg^2+^-free artificial seawater (449 mM NaCl, 9 mM KCl, 33 mM Na_2_SO_4_, 2.15 mM NaHCO_3_, 10 mM Tris-HCl, 2.5 mM EGTA, pH 8.0). Tentacles were cut away, as they secrete a lot of mucus (which affected the quality of the isolated DNA). The remaining stalks and the bases of individual *Xenia* colonies were placed in 100 μl DNAzol (Invitrogen) in a 1.5-ml microcentrifuge tube. The tissues were cut into small pieces by a scissor to make fragment sizes of about 1/10th of the original size. These fragments were further minced by a small pestle made for 1.5-ml microcentrifuge tubes (Fisher Scientific, 12-141-364). Then, 900 μl DNAzol was added, followed by vortexing the sample and then transferred to a 15-ml conical tube. Four millilitres of DNAzol and 50 μl of 10 mg/ml RNase A were then added to the tube and mixed, followed by incubation at 37 °C for 10 min. Then, 25 μl of 20 mg/ml proteinase K was then added, mixed and the tube was incubated at 37 °C for another 10 min. The sample was centrifuged at 5,000*g* for 10 min. The supernatant was transferred to another 15-ml tube. After adding 2.5 ml ethanol, the tube was gently mixed by inverting several times. The tube was left to stand at room temperature for 3 min followed by centrifugation at 1,000*g* for 10 min to pellet the DNA. The supernatant was discarded and the DNA pellet was resuspended in 500 μl TE (10 mM Tris-HCl, 1 mM disodium EDTA, pH 8.0). After the DNA had dissolved, 500 μl of phenol:chloroform:isoamyl alcohol (25:24:1) was added, and the tube was placed on the Intelli-Mixer RM-2S for mixing using programme C1 at 35 rpm for 10 min. The mixture was then transferred to a 2-ml phase-lock gel (QuantaBio, Cat. 2302820) and centrifuged at 4,500 rpm for 10 min. The aqueous phase was transferred into a new 2-ml tube, 200 μl 5 M ammonium acetate and 1.5 ml ice-cold ethanol were added followed by centrifugation at 10,000*g* for 10 min to pellet DNA. The pellet was washed twice with 1 ml 80% ethanol. After removing as much ethanol as possible, the DNA pellet was left to dry at 42 °C for 1 min, and then resuspended in 50 μl TE buffer.

### Illumina sequencing

Genomic DNA prepared as in ‘Genomic DNA isolation from *Xenia* sp.’ was fragmented into about 400 bp, and libraries were made with ThruPLEX DNA-Seq kit (TaKaRa) according to the manufacturer’s manual. These libraries were sequenced using the NEXseq500 platform with NextSeq 500/550 High Output Reagent Cartridge v2 (Illumina).

### Nanopore sequencing

Genomic DNA was used to build Nanopore sequencing libraries with Ligation Sequencing Kit (SQK-LSK108, Oxford Nanopore Technologies), following the manufacturer’s manual. For the first three runs, genomic DNA was not fragmented, to generate long reads. To obtain more reads, for the fourth run of Nanopore sequencing, genomic DNA was sheared to 8–10 kilobases by g-TUBE (Covaris, 520079). The libraries were sequenced in R9.4.1 flow cells on a MinION device (Oxford Nanopore Technologies). MinKNOW (v.1.7.3) was used to collect raw signal and Albacore (v.2.3.3) was used for base-calling. All the data were combined for genome assembly.

### Hi-C

To perform Hi-C on *Xenia* sp. tissue, we modified a previously published protocol for nuclear in situ ligation^13^, as described in detail.

#### Fix and dissociate tissues (step 1)

Eight polyps (about 10^8^ cells) were fixed with 4% paraformaldehyde (PFA) overnight. After washing twice with 3.3× PBS^38^ and dissociating the tissue in 2 ml 3.3× PBS using a 7-ml glass Dounce tissue grinder (Wheaton), another 3 ml 3.3× PBS was added. The mixture was then transferred to a 15-ml conical tube and centrifuged at 1,000*g* for 3 min (Sorvall Lynx 6000 centrifuge, ThermoFisher Scientific). The pellet was washed once with 5 ml 3.3× PBS.

#### Nuclear permeabilization and chromatin digestion (step 2)

The pellet from step 1 was resuspended in 10 ml ice-cold Hi-C lysis buffer (10 mM Tris, pH 8.0, 10 mM NaCl, 0.2% NP-40, 1× protease inhibitors cocktail (Roche, 04693132001)) and rotated for 30 min at 4 °C followed by centrifugation at 1,000*g* for 5 min at 4 °C. The pellet was resuspended with 1 ml ice-cold 1.2× NEB3.1 (120 μl NEB3.1 to 880 μl ddH_2_O) buffer and transferred to a 1.5-ml microcentrifuge tube followed by centrifugation at 1,000*g* for 5 min at 4 °C. The pellet was washed again with 1 ml ice-cold 1.2× NEB3.1 followed by centrifugation. After removing the supernatant, 400 μl 1.2× NEB3.1 buffer and 12 μl of 10% SDS were added to the pellet. P200 pipette tip was used to thoroughly resuspend and dissociate the pellet. The mixture was then incubated at 65 °C for 10 min at 950 rpm in a Thermomixer (Eppendorf). After cooling the mix on ice for 5 min, 40 μl 20% Triton X-100 was added to the mixture to neutralize the SDS. After carefully mixing by pipetting with a P200 pipette tip and inverting the tube several times, the mixture was then incubated at 37 °C for 60 min with rotation (950 rpm) in a Thermomixer. To digest the crosslinked genomic DNA, 30 μl of 50 U/μl BglII (NEB R0144M) was added to the mixture and incubated overnight at 37 °C with rotation at 950 rpm in a Thermomixer.

#### Fill in 5′ overhang generated by BglII digestion with biotin (step 3)

A nucleotide mix containing dATP, dGTP and dTTP was made by adding 1 μl each of 100 mM dATP, dGTP and dTTP into 27 μl ddH_2_O. To the 480.0 μl BglII-digested nuclear preparation from the above step 2, 4.5 μl of the nucleotide mix, 15 μl 1 mM biotin-16-dCTP (Axxora, JBS-NU-809-BIO16) and 10 μl 5 U/μl Klenow (NEB, M0210L) were added followed by incubation at 37 °C for 90 min with intermittent gentle shaking at 700 rpm for 10 s after every 20 s using Thermomixer. The tube was also taken out and inverted every 15–20 min. After this incubation, the mixture was kept on ice.

#### Proximity ligation (step 4)

The mixture from step 3 was transferred to a 50-ml conical tube followed by adding 750 μl 10× T4 ligase buffer (NEB B0202S, no PEG), 75 μl 100× BSA (NEB), 6,140 μl water, 25 μl 30 U/μl T4 DNA ligase (Thermo Scientific, EL0013), and incubating at 16 °C overnight.

#### Reverse crosslink and DNA isolation (step 5)

To the reaction mixture from step 4, 25 μl of 20 mg/ml proteinase K (Invitrogen, 25530-049) was added and the mixture was divided equally into 8×1.5-ml microcentrifuge tubes (about 950 μl per tube). The tubes were then incubated overnight at 65 °C with rotation at 950 rpm in a Thermomixer. The next day, 3 μl 20 mg/ml proteinase K was added to each tube followed by incubation at 65 °C for 2 h with mixing in Thermomixer. The mixtures were combined into one 50-ml conical tube. After cooling down to room temperature, 10 ml phenol (pH 8.0) (Sigma) was added and mixed by vortex for 2 min. The mixture was then centrifuged for 10 min at 3,000*g* (Sorvall Lynx 6000 centrifuge). The supernatant containing the DNA was mixed with 10 ml phenol:chloroform (1:1) (pre-warmed to room temperature) and vortexed for 2 min. The whole mixture was then transferred to a 50-ml MaXtract High Density tube (Qiagen, 129073) and centrifuged at 1,500*g* for 5 min (Sorvall Lynx 6000 centrifuge). The top phase containing the Hi-C DNA was transferred to a 50-ml conical tube and the volume (usually about 10 ml) was adjusted to 10 ml with 1× TE as needed. To pellet the DNA, 1 ml 3 M Na-acetate, 5 μl 15 mg/ml GlycoBlue (Invitrogen AM9515) and 10 ml isopropanol were added to the mixture and incubated at −80 °C for >1 h. The DNA was then pelleted by centrifugation at 17,000*g* for 45 min at 4 °C (Sorvall Lynx 6000 centrifuge). The Hi-C DNA pellet was resuspended in 450 μl 1× TE and transferred to a 1.5-ml microcentrifuge tube followed by adding 500 μl phenol:chloroform (1:1). After mixing by vortex, the mix was centrifuged at 18,000*g* for 5 min at room temperature. The top aqueous layer was collected into another tube followed by adding 40 μl 3M Na-acetate, 1 μl 15 mg/ml GlycoBlue (Invitrogen AM9515, 300 μl) and 1 ml ice-cold 100% ethanol. After incubating at −80 °C for >30 min, the DNA was centrifuged at 21,000*g* for 30 min at 4 °C. The DNA pellet was washed with freshly prepared 70% ethanol and air-dried, followed by dissolving in 45 μl EB (10mM Tris, pH 8.0). The contaminated RNA in the DNA preparation was digested by adding 0.5 μl 10 mg/ml RNaseA and incubated at 37 °C for 30 min.

#### Remove biotin from the free DNA (unligated DNA) ends (step 6)

To remove the biotin at the free DNA ends, 1.0 μl 10 mg/ml BSA (NEB, 100×), 10 μl 10× NEB 2.1 buffer, 1 μl 10 mM dATP, 1 μl 10 mM dGTP and 5 μl T4 DNA polymerase (NEB M0203S), and 42 μl water were added to 40 μl (about 3 μg) Hi-C DNA preparation from step 5. The mixture was divided into two equal aliquots in 2 PCR tubes and incubated at 20 °C for 4 h. Then, 2 μl of 0.5 M EDTA was added to each of the two tubes to stop the reaction. The Hi-C DNA was then purified using the Clean and Concentrator Kit (ZYMO, D4013) followed by elution with 50 μl EB.

#### Biotin pull-down of DNA and second DNA digestion (step 7)

In brief, 60 μl of Dynabeads MyOne Streptavidin C1 (Invitrogen) was washed in 1.5-ml non-sticking microcentrifuge tubes (Ambion) with 200 μl 2× binding buffer (10 mM Tris, pH 8, 0,1 mM EDTA, 2 M NaCl) twice, followed by resuspension in 50 μl 2× binding buffer. The 50 μl Hi-C DNA from step 6 was added followed by rotating for 30 min using Intelli-Mixer (ELMI) at room temperature. The beads were collected using a magnetic stand and washed with 100 μl 1× binding buffer followed by washing with 100 μl 1× NEB4 buffer twice and resuspending in 50 μl 1× NEB4 buffer. The DNA on beads was digested using 1 μl 10 U/μl AluI (NEB, R0137S) at 37 °C for 60 min. The beads were collected on a magnetic stand followed by washing with 100 μl 1× binding buffer, and then 100 μl EB. The beads were resuspended in 30 μl EB.

#### A-tailing (step 8)

The 30-μl beads from step 7 were mixed with 5 μl NEB Buffer 2, 10 μl 1 mM dATP, 2 μl H_2_O, 3 μl Klenow (3′–5′ exo-) (NEB M0212L) and incubated at 37 °C for 45 min. After the reaction, the beads were collected by a magnetic stand followed by washing with 100 μl 1× binding buffer and then 100 μl EB. The beads were resuspended in 50 μl EB.

#### Sequencing adaptor ligation (step 9)

The 50-μl beads from step 8 was mixed with 3.75 μl sequencing adaptor (TruSeq RNA Sample Prep Kit v.2), 10 μl 1× T4 DNA ligase buffer, 3 μl T4 DNA Ligase (30 U/μl) (Thermo Scientific, EL0013) and incubated at room temperature for 2 h. The beads were collected by a magnetic stand followed by washing twice with 400 μl 1× binding buffer + 0.05% Tween, 200 μl 1× binding buffer, and then 100 μl EB. The beads were resuspended in 40 μl EB. To release the DNA from the beads, the mixture was incubated at 98 °C for 10 min and then centrifuged at 500 rpm to pellet the streptavidin beads.

#### Sequencing library preparation (step 10)

TruSeq RNA Library Prep Kit was used to make DNA sequencing library (eight PCR cycles were used) and the DNA was sequenced by NextSeq 500.

### scRNA-seq

For each of the six scRNA-seq library preparation, 1 polyp, 8 tentacles, and 2 stalks or 2 regenerating stalks of *Xenia* sp. were dissociated into single cells in 1 ml digestion buffer, containing 3.6 mg/ml dispase II (Sigma, D4693), 0.25 mg/ml liberase (Sigma, 5401119001), 4% l-cysteine in Ca^2+^-free seawater (393.1 mM NaCl, 10.2 mM KCl, 15.7 mM MgSO_4_·7H_2_O, 51.4 mM MgCl_2_·6H_2_O, 21.1 mM Na_2_SO_4_, and 3 mM NaHCO_3,_ pH 8.5) and incubated for 1 h at room temperature. After digestion, fetal bovine serum was added to a final concentration of 8% to stop enzymatic digestion. The cell suspension was filtered through a 40-μm cell strainer (FALCON). A low concentration (0.1 μg/ml) of DAPI that can only be taken up by dead cells was used to measure cell viability. Only cell suspensions in which more than 90% of cells that did not take up DAPI were used. Cells were counted by haemocytometer and diluted with the same 4% l-cysteine in Ca^2+^-free seawater used in the digestion buffer into 1,000 cells per μl. Around 17,000 cells per sample were used for single-cell library preparation using the 10x Genomics platform with Chromium Single Cell 3′ Library and Gel Bead Kit v.2 (PN-120267) (v.2 chemistry) or Chromium Next GEM Single Cell 3′ GEM, Library and Gel Bead Kit v.3.1 (PN-1000121, v.3 chemistry), Single Cell 3′ A Chip Kit (PN-1000009) or Chromium Next GEM Chip G Single Cell Kit (PN-1000127), and i7 Multiplex Kit (PN-120262). For the scRNA-seq library construction, we followed the 10x protocol exactly. In brief, for v.2 chemistry, 17.4 μl cell suspension and 16.4 μl nuclease-free water were mixed with 66.2 μl reverse transcription master mix. Of this 100 μl mix, 90 μl was loaded into the chip provided in the Single Cell 3′ A Chip Kit. For v.3 chemistry, 16.5 μl cell suspension and 26.7 μl nuclease-free water were mixed with 31.8 μl reverse transcription master mix. Of this 75 μl mix, 70 μl was loaded into the Chromium Next GEM Chip G. After barcoding, cDNA was purified and amplified with 11 PCR cycles. The amplified cDNA was further purified and subjected to fragmentation, end repair, A-tailing, adaptor ligation and 14 cycles of sample index PCR. Libraries were sequenced using Illumina NextSeq 500 for paired-end reads. Read 1 is 26 bp (v.2 chemistry) or 28 bp (v.3.1 chemistry) and read 2 is 98 bp.

In our initial scRNA-seq using the 10x Genomics v.2 chemistry, we obtained fewer unique molecular identifiers (UMIs) (median number, 801) and genes (median number, 467) per *Xenia* cell compared to other model organisms, such as the mouse thymus^39^ (median UMI 5,802 and median gene number 2,178), but higher than in *Nematostella*^18^ (median UMI 541 and median gene number 278). The new and improved v.3 chemistry substantially improved our scRNA-seq. We captured more cells per library (v.3 7,874 versus v.2 2,883), a higher number of median genes per cell, (v.3 943 versus v.2 467) and median UMI per cell (v.3 2,027 versus v.2 801). Our v.3 dataset has lower quality than those of the mouse thymus^39^ and *Hydra*^40^ scRNA-seq datasets (Supplementary Table 1). This suggests that, even using v.3 chemistry, the presence of seawater and/or *Xenia*-sp.-specific features may contribute to the reduced scRNA-seq quality.

We noticed the mapping rate in v.3 chemistry is lower than in v.2 chemistry. We sequenced more reads for the v.3 libraries, because v.3 captured more total cells and more RNA molecules per cell. Although we sequenced more for the v.3 libraries, we obtained lower sequence saturation (on average, 79.6% in v.3 libraries and 92.6% in v.2 libraries). Because the v.2 and v.3 reagent contents are proprietary information, it is difficult for us to assess why the two methods gave different results. Regarding our library preparation, the v.3 method entailed 22% of the total volume coming from the cell suspension in the Ca^2+^-free seawater, while in the v.2 method, 17.4% of the total volume came from the Ca^2+^-free seawater cell suspension. We therefore know that one difference between the two methods is that the salt concertation in v.3 library preparation is higher than that in the v.2 library preparation. The higher salt concentration in v.3 could lead to a higher RNA extraction efficiency in the v.3 library preparation, which could contribute to the difference between our v,2- and v,3-based scRNA-seq. Althought the 10x platform worked well for the *Xenia* sp. we studied here, it is important to keep in mind that modifications may be needed for successful scRNA-seq for other marine cnidarians.

### Quantification of endosymbiotic *Xenia* cells by microscopy and FACS

To quantify the endosymbiotic cell percentage in *Xenia*, we first applied a microscopy-based strategy. By imaging cryo-preserved tissue sections stained with 1 μg/ml DAPI that labelled all nuclei, we determined the total number of *Xenia* cells per section by counting the number of *Xenia* cell nuclei: these nuclei are easily differentiated from the alga nuclei when overlapped with the autofluorescence signal in far red channel from algae. The number of *Xenia* cells containing alga is estimated by counting the number of algae surrounded by *Xenia* tissue. The estimated percentage by this method is on average 2–6%, depending on whether the sections were taken from stalks or tentacles (Extended Data Fig. 4d). The limitation of this method is that some algae that appear to be inside the tissue may be between *Xenia* cells and not inside cells. Therefore, this estimate could represent an upper limit of the percentage of alga-containing *Xenia* cells.

In the second method, we used FACS to separate free algae and algae contained inside the *Xenia* cells. *Xenia* polyps were dissociated into single-cell suspension with the same preparation method as described in ‘scRNA-seq’. The cells were fixed with 1% (final concentration) formaldehyde on ice for 1 h, followed by 0.2% Triton X-100 permeablization and 1 μg/ml DAPI staining. We first separated free algae and alga-containing *Xenia* cells according to the algae autofluorescence in the Cy5.5 channel. Free algae and algae inside *Xenia* cells should have different forward scatter (FSC) and side scatter (SSC) signals because the alga inside *Xenia* cells is enclosed by the *Xenia* cellular membrane structure. Thus, we used FSC and SSC to further gate the total population of algae into two subpopulations. Microscopy analyses showed that this gating separated free algae and alga-containing *Xenia* cells. To determine the total *Xenia* cell number, *Xenia* cells together with algal cells were gated according to DAPI-positive signal followed by gating with the Cy5.5 signal. The total *Xenia* cells were calculated as alga-free *Xenia* cells plus the alga-containing *Xenia* cells. On the basis of these FACS analyses, we were able to estimate the percentage of alga-containing *Xenia* cells in *Xenia* polyps to be about 2% of total *Xenia* cells. The illustration of this FACS sorting can be found in Extended Data Fig. 7b–g. Because the procedure of single-cell dissociation may cause an alga-containing *Xenia* cell to lose its alga, the approximately 2% of alga-containing *Xenia* cells obtained by the FACS method probably represents an underestimation. Thus, we estimate the fraction of alga-containing *Xenia* cells to be about 2–6%.

### Bulk RNA-seq

Total RNA was isolated from 3 polyps, 32 tentacles or 6 stalks by RNeasy Plus Mini Kit (Qiagen). To obtain additional transcriptomes from different cell types, we dissociated coral tissue into individual cells according to a previously published method^41^ and subjected the dissociated cells to OptiPrep-based cell separation^42^. Cells with different densities were separated into four layers, and RNA was isolated from each layer with RNeasy Plus Mini Kit (Qiagen). For transcriptome of FACS-isolated alga-containing and alga-free cells, three polyps were dissociated with the same protocol as used in the scRNA-seq and the dissociated cells were subjected to FACS. Cy5.5-positive and -negative cells were collected as alga-containing and alga-free cells, respectively, and used for total RNA extraction as above. cDNA libraries were built according to TruSeq Stranded mRNA Library Prep Kit (Illumina) and subjected to Illumina NextSeq 500 for sequencing. For gene annotation, paired-end sequencing of 75 bp for each end was used. For FACS-isolated bulk-cell transcriptomes, single-end sequencing of 75 bp was used.

### *Xenia* regeneration, BrdU labelling and EdU pulse–chase

Individual *Xenia* sp. polyps were placed into a well of 24-well cell-culture plate (Corning) containing 1 ml artificial seawater from our aquatic tank. The polyps were allowed to settle in the well for 5–7 days before cutting away the tentacles. After cutting, there were a lot algae released into the seawater, which together with the free algae living inside the cavity of the coral could serve as alga reservoirs for the uptake of algae during regeneration.

For the BrdU labelling experiments, 0.5 mg/ml BrdU was added into the well 2 d before sample collection. The BrdU-labelled stalks were fixed by 4% PFA overnight, followed by washing with PBST (PBS+0.1% Tween 20) twice for 10 min each. The stalk was then balanced with 30% sucrose overnight followed by embedding in OCT, frozen in dry ice bathed in ethanol and subjected to cryo-sectioning. The slides were washed with PBS 3 times for 5 min each time followed by treating with 2 M HCl containing 0.5% Triton X-100 for 30 min at room temperature. The slides were then incubated with PBST (0.2% Triton X-100 in PBS) 5 min for 3 times each followed by blocking with 10% goat serum and then incubating with mouse anti-BrdU antibody (ZYMED, 18-0103, 1:200 dilution in 10% goat serum) at 4 °C overnight. Slides were washed with PBST 3 times for 10 min each followed by incubation with the secondary antibody (Invitrogen) for 1 h at room temperature and washing with PBST 3 times for 10 min each. The nuclei were counterstained with Hoechst 33342 and the signal was visualized using a confocal microscope (Leica). Clear BrdU signal in the nucleus labelled by Hoechst was counted as a BrdU^+^ cell. If the *Xenia* BrdU^+^ nucleus was juxtaposed to an alga, it was counted as an alga-containing BrdU^+^ *Xenia* cell.

For EdU pulse–chasing experiments, the regenerating *Xenia* stalks were incubated with 1 mM EdU during regeneration day 3 and day 4. After washing out EdU, the coral was incubated with artificial seawater and samples were collected on regenerating days 7, 9, 11, 13, 15, 17 and 19. The samples were dissociated into single-cell suspensions followed by fixing with 1% formaldehyde at 4 °C overnight as described in ‘scRNA-seq’. The fixed cells were pelleted at 800*g* for 5 min and further fixed with 4% PFA for two days to block the autofluorescence in the 488-nm channel. Then, the EdU click chemistry was carried out using the Click-iT EdU Cell Proliferation Kit (Invitrogen, C10337) according to manufacturer’s protocol. The cells were further stained with DAPI, and then analysed by FACS as described in Extended Data Fig. 7 and ‘Quantification of endosymbiotic *Xenia* cells by microscopy and FACS’.

### Whole-mount RNA ISH

To perform RNA ISH on *Xenia*, we modified the whole-mount RNA ISH protocol for zebrafish^43^.

For making gene-specific sense or anti-sense probes, we designed primers (Supplementary Table 7) to genes of interest for PCR to amplify gene fragments from *Xenia* sp. cDNA. The T3 promoter sequence was added to the 5′ of the reverse primers so that the PCR products could be directly used for synthesizing anti-sense RNA probes by T3 RNA polymerase (Promega, P2083) using DIG RNA Labelling Mix (Roche, 11277073910). DIG-labelled RNA probes were purified by RNA Clean and Concentrator-5 (ZYMO), heated to 80 °C for 10 min, immediately transferred on ice for 1 min, and then diluted in Prehyb^+^ buffer (50% formamide, 5× saline–sodium citrate buffer (SSC, 0.75M NaCl, 0.075M sodium citrate), 50 μg/ml heparin, 2.5% Tween 20, 50 μg/ml single-stranded DNA (Sigma, D1626)) to a final concentration of 0.5 μg/ml, and stored at −20 °C until use.

*Xenia* polyps were relaxed in Ca^2+^-free seawater for 30 min and then fixed in 4% PFA in Ca^2+^-free seawater overnight at 4 °C. Fixed polyps were washed with PBST (0.1% Tween 20 in PBS) twice for 10 min each, and then incubated in 100% methanol at −20 °C overnight.The next day, the tissues were washed sequentially in 75%, 50% and 25% methanol for 5 min each and then washed in PBST for 10 min. They were then treated with 50 μg/ml proteinase K in PBST for 20 min followed by a brief wash in PBST. The tissues were post-fixed in 4% PFA at room temperature for 20 min and then washed with PBST 2 times for 10 min each. Prehybridization was performed in Prehyb^+^ at 68 °C for 2 h, followed by incubation with probes in Prehyb^+^ overnight at 68 °C. To probe gastrodermis markers, 2% SDS (final concentration) was added to help the probes to penetrate the tissue. After probes were removed, samples were washed sequentially in 2× SSC (0.3 M NaCl and 0.03 M sodium citrate) containing 50% formamide for 20 min twice, 2× SSC containing 25% formamide for 20 min, 2× SSC for 20 min twice, and 0.2× SSC for 30 min 3 times each, all at 68 °C. Then, samples were washed in PBST at room temperature for 10 min and incubated in DIG blocking buffer (1% ISH blocking reagent (Roche, 11096176001) in maleic acid buffer (0.1 M maleic acid, 0.15 M NaCl, pH 7.5) for 1 h at room temperature, followed by incubation in anti-DIG antibody (anti-digoxigenin-AP (Roche, 11093274910)) at 1:5,000 dilution in DIG blocking buffer overnight at 4 °C. The next day, the samples were washed in PBST for 10 min 3 times each at room temperature, then in 9.5T buffer (100 mM Tris-HCl pH 9.5, 50 mM MgCl_2_, 100 mM NaCl, 0.1% Tween 20) for 10 min 3 times each at room temperature. Hybridization signals were revealed by incubation in BCIP/NBT buffer (1 SIGMAFAST BCIP/NBT tablet (Sigma, B5655) in 10 ml H_2_O)) at 4 °C until brown–purplish colours were sufficiently dark. For this study, the colour development took 48 h. The samples were then washed in PBST twice for 10 min each. The samples were post-fixed in 4% PFA overnight at 4 °C, followed by washing in PBST twice for 10 min each, and then washed in methanol for 3 h at room temperature. The tissues were kept in PBS and imaged using SMZ1500 microscope (Nikon) under Ring Light System (Fibre-Lite). For cross- sections of stalks, the whole-mount sample was processed for cryo-section as described in ‘*Xenia* regeneration, BrdU labelling and EdU pulse–chase’.

### RNAscope ISH assay for *LePin* and *Granulin 1* expression

To visualize RNA expression in endosymbiotic cells, we used the ultrasensitive RNAscope ISH approach (Advanced Cell Diagnostics (ACD)). *LePin*- *or Granulin-1*-specific oligonucleotide probes were ordered from ACD (see Supplementary Table 7 for further information). The fluorescent RNAscope assay was carried out by RNAscope Multiplex Fluorescent Reagent Kit v.2 (ACD) according to the manufacturer’s protocol. The chromogenic assay was carried out by RNAscope 2.5 HD Duplex Detection Kit (ACD), according to manufacturer’s protocol. Both assays used the cryo-section of the fixed *Xenia* polyp prepared according to the manufacturer’s protocol.

### Genome assembly

Sequencing data from Nanopore were used to initiate the genome assemble by Canu (v.1.7)^44^. The assembled genome was further polished with Illumina short reads by Nanopolish (v.0.9.2, https://github.com/jts/nanopolish) with 5 cycles, which resulted in 1,482 high-quality contigs for the diploid genome. The diploid genome assembly was separated into haploid by HaploMerger2^45^. The haploid genome assembly was further subject to Hi-C assisted scaffolds by 3D de novo assembly pipeline, Juicer (v.1.5)^14^. By aligning all the Illumina genomic sequencing data with the assembled genome, we found 0.45% single nucleotide polymorphism (SNP) within the whole assembled genome of the *Xenia* sp.

### Gene annotation

The funannotate genome annotation pipeline (v.1.3.3, https://github.com/nextgenusfs/funannotate) was used to annotate the *Xenia* sp. genome. In brief, transcriptome data were assembled by Trinity (v.2.6.6)^46^ and used to generate the gene models based on the presence of mRNA by PASApipeline (v.2.3.2)^47^. These gene models were used as training sets to perform de novo gene prediction by AUGUSTUS (v.3.2.3)^48^ and GeneMark-ES Suite (v.4.32)^49^. All gene models predicted by PASApipeline, AUGUSTUS and GeneMark were combined and subjected to EVidenceModeller to generate combined gene models^50^. The predicted genes were filtered out if more than 90% of the sequence overlapped with repeat elements as identified by RepeatMasker and RepeatModeler (http://www.repeatmasker.org). PASA was further used to add 3′ and 5′ untranslated region sequences to the remaining predicted genes. Pfam (v.31.0), Interpro (v.67.0), Uniprot (v.2018_03), BUSCO (v.1.0)^51^ databases and eggnog-mapper (v.1.3)^52^ were used to annotate the function of these gene models. Among all the predicted genes, 23,939 (82.5%) gene models were supported by transcriptome data because they have detectable reads (reads number >0). Among these models, 20,397 have read numbers >5.

### Phylogeny tree analysis

We used OrthoFinder (v.2.2.7) to find orthologues from different species on the basis of protein sequences from 13 species listed Fig. 1d, and inferred the species tree^53,54^. In brief, ‘orthofinder -S diamond -t 22 -M msa -f fasta_files’ was used to generate the result. Diamond (v.0.9.21) was used for sequence search and OrthoFinder grouped 308,348 genes (83.8% of total) into 19,244 orthogroups. One thousand six hundred and one orthogroups, according to previously reported method^55^, with a minimum 10 species having single-copy genes, were used to infer the species tree. These orthogroups were subjected to multiple sequence alignment by MAFFT (v.7.407) and columns with more than eight gaps were trimmed. The trimmed alignment with 73.6% data occupancy (see Source Data for Fig. 1d) was used to infer the maximum likelihood unrooted species tree by FastTree (v.2.1.10) with the default configuration in OrthoFinder. This species tree was further rooted by the STRIDE algorithm, which has been demonstrated to correctly root the species tree spanning a wide range of time scales and taxonomic groups^56^.

### Single-cell clustering and marker gene identification

The raw single-cell sequencing data were de-multiplexed and converted to FASTAQ format by Illumina bcl2fastaq (v.2.20.0) software. Cell Ranger (v.3.1.0, https://support.10xgenomics.com/single-cell-gene-expression/software/overview/welcome) was used to de-multiplex samples, process barcodes and count gene expression. The sequence was aligned to the annotated *Xenia* sp. genome and only the confidently mapped and non-PCR duplicated reads were used to generate gene expression matrix for each library with ‘cellranger count’ command. The expression matrix of Cell-Ranger-identified cells from each library was read into R and further analysed with Seurat (v.3.0.2)^57^. Cells with UMI numbers less than 400 or mitochondria gene expression >0.2% of total reads were excluded for downstream analysis. To further remove outliers, we calculated the UMI number distribution detected per cell and removed cells in the top 1% quantile. To remove batch effect and integrate data from different libraries, we applied the Seurat v.3 method for data integration^57^. For each dataset, we identified the top 1,000 genes with the highest dispersion. We used the top 1,000 genes in the non-regeneration sample as anchor features to identify anchors between different non-regeneration datasets. The first 20 dimensions were used to generate the integrated data. Dimensional reduction was carried out on the integrated data, and used for further clustering analysis. Clustering and marker gene identification in non-regeneration condition was further performed with Seurat v.3. The cell clusters in regeneration samples were identified with the label transfer method in Seurat v.3. All violin plots were generated using Seurat VlnPlot function.

### Identification of *Xenia* sp. cells performing endosymbiosis with Symbiodiniaceae

The bulk transcriptome data of FACS-isolated alga-containing or alga-free *Xenia* cells were aligned to *Xenia* sp. genome by STAR (v.2.5.3a)^58^. Individual gene expression (reads per kilobase of transcript, per million mapped reads) for each sample were calculated by RSEM (v.1.3.0)^59^. The gene-expression levels of each bulk RNA-seq of FACS-isolated cells were compared with the gene-expression levels calculated using average UMI number for each gene in each cell cluster identified by scRNA-seq. The Pearson correlation coefficient was calculated for each comparison.

### Pseudotime analysis

To infer the trajectory of endosymbiotic *Xenia* cells, we integrated scRNA-seq data of regenerating and non-regenerating samples using Seurat v.3. All cells belonging to the endosymbiotic cell cluster (cluster 16, total of 382 cells) were subjected to Monocle (v.2.10.1)^29^ analyses. To find the variable genes among these cells for downstream analysis, we grouped these cells into three subclusters with Monocle clusterCells function (with default setting for most parameters, except for num_clusters = 4, which generated 3 clusters). Each of these three subclusters contains 247, 53 or 82 cells. The top 1,000 differentially expressed genes between these three subclusters were used as ordering genes to construct the trajectory by DDRTree algorithm. The differentially expressed genes along pseudotime were detected using the differentialGeneTest function in Monocle. The cell numbers in each of the five predicted endosymbiotic cell states are state 1 = 36, state 2 = 109, state 3 = 155, state 4 = 45 and state 5 = 37.

### RNA velocity

RNA velocity estimation was carried out using the velocyto.R program (http://velocyto.org, v.0.6), according to the instructions^30^. In brief, velocyto used raw data of the regeneration sample to count the spliced (mRNA) and unspliced intron reads for each gene to generate a .loom file. This .loom file was loaded into R (v.3.6.1) using the read.loom.matrices function and used to generate the RNA velocity map. The RNA velocity map was projected into the *t*-SNE space that was identified by Seurat.

### Reporting summary

Further information on research design is available in the Nature Research Reporting Summary linked to this paper.

## Online content

Any methods, additional references, Nature Research reporting summaries, source data, extended data, supplementary information, acknowledgements, peer review information; details of author contributions and competing interests; and statements of data and code availability are available at 10.1038/s41586-020-2385-7.

## Supplementary information

Reporting Summary

Supplementary Table Supplementary Table 1: Summary of *Xenia* scRNA-seq libraries and comparisons of scRNA-seq datasets. Six *Xenia* scRNA-seq libraries based on v2 or v3 chemistry are listed with total reads (Total_reads_No), percentage of reads mapped to genome, total cell number identified, mean unique molecular identifier (mean UMI), genes detected per cell (Ngenes_per_cell), UMI percentile at 10, 50 or 90 are shown. The median UMI number, median gene number, and average cell number of the *Xenia* scRNA-seq libraries based on v2 or v3 chemistry were compared to that of scRNA-seq of *Nematostella*, *Hydra*, and mouse.

Supplementary Table Supplementary Table 2: Genes that characterize each of the 16 *Xenia sp*. cell clusters. The columns show: Gene_ID, Symbol, Cluster (markers for which cluster), p_val_adj (Bonferroni adjusted p-values), avg_logFC (the log-fold change of the average expression between a cluster and other clusters), specificity_within_cluster (percentage of cells within the cluster having higher expression than the average of gene expression in all cells), specificity_outside_cluster (percentage of cells outside of the cluster having higher expression than the average expression in all cells), Best_hit_in_human (the best blast hits in human genome with e value less than 10^-5^, hits name consists of Uniprot accession id and gene name), domain information (identified from NCBI Conserved Domains Database), and protein sequences are shown. Cell numbers in 1-16 cell clusters: n= 2,794; 2,704; 2,073; 1,679; 1,511; 1,374; 1,248; 1,069; 986; 923; 797; 649; 575; 321; 246; 185; respectively.

Supplementary Table Supplementary Table 3: Bulk transcriptomes of the FACS-isolated *Xenia* cells. FACS-isolated algae-containing and algae-free cells were subjected for RNA-seq. Two biological replicates were obtained. The gene expression level is calculated in Reads Per Kilobase of transcript per Million mapped reads (RPKM).

Supplementary Table Supplementary Table 4: The top 89 marker genes found in the endosymbiotic *Xenia sp*. cells. The columns show: Gene ID; Symbol (gene symbol identified by the gene annotation pipeline); Name (gene names we provided according to their homolog in other species or according to their predicted domains); Adjusted p value (Bonferroni adjusted p-values, significance level of the identified genes in the scRNA-seq as markers for the endosymbiotic cells); Specificity_within_cluster (percentage of cells within the cluster having higher expression than the average of gene expression in all cells); Specificity_outside_cluster (percentage of cells outside of the cluster having higher expression than the average expression in all cells); Best hits in human (the best blast hits in human genome with e value less than 10^-5^, hits name consists of Uniprot accession id and gene name); Domain or Superfamily (domains were identified using NCBI Conserved Domains Database (CDD)); Comparison to other species (manual annotation by comparing each gene against the ncbi nr database). The best hits in human often only involve a specific region of a gene.

Supplementary Table Supplementary Table 5: Expression levels of the 89 cluster 13 marker genes in the FACS isolated algae-containing or algae-free *Xenia sp*. cells. Gene expression level is calculated in Reads Per Kilobase of transcript per Million mapped reads (RPKM).

Supplementary Table Supplementary Table 6: Differentially expressed genes along the cell lineage trajectory that help to define the five states of the endosymbiotic *Xenia* cells. The columns show: Gene ID; Symbol (gene symbol identified by the gene annotation pipeline); Enriched_in_state (genes enriched in the indicated state). The relative expression, defined by genSmoothCurves function from monocle, of each gene in the five states are listed. Best hits in human (the best blast hits in human genome with e value less than 10^-5^, hits name consists of Uniprot accession id and gene name); Domain (domain information identified from NCBI Conserved Domains Database). Reference (list relevant references for the genes with known functions in regulating oxidative stress, highlighted in green and they are preferentially expressed in state5 cells). The references are listed next to each of these gene.

Supplementary Table Supplementary Table 7: RNA *in situ* hybridization probes. The list contains the sense and anti-sense probes for the whole mount RNA ISH and the catalog numbers for probes used in RNAscope ISH.

Video 1 A video of the laboratory *Xenia* sp. in our aquarium.

## Source data

Source Data Fig. 1

Source Data Fig. 3

Source Data Extended Data Fig. 4

Source Data Extended Data Fig. 6

Source Data Extended Data Fig. 7

## Figures and Tables

**Extended Data Table 1:**
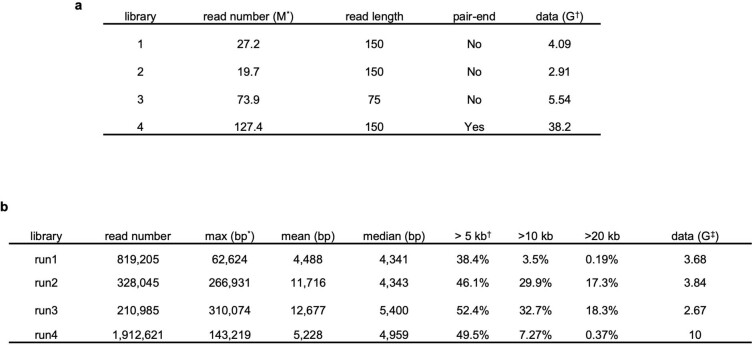
Summary of sequencing libraries for genome assembly

**a**, Summary of Illumina sequencing for genome assembly. The table shows sequence information from four library preparations from four *Xenia* colonies for Illumina sequencing. Read number indicates the total number of reads obtained. Read length indicates the individual read length, by paired-end sequencing. Data indicate total sequence data in gigabases. M*, million; G^†^, gigabase.

**b**. Summary of Nanopore sequencing for genome assembly. Statistics of all sequence information from four different runs from four *Xenia* colonies of Nanopore sequencing, including maximum, mean and median read-length statistics, and the percentages of reads that have bigger sizes than the indicated number: >5 kb, >10 kb, and >20 kb. bp*, base pair; kb^†^, kilobase; G^‡^, gigabase.

**Extended Data Table 2:**
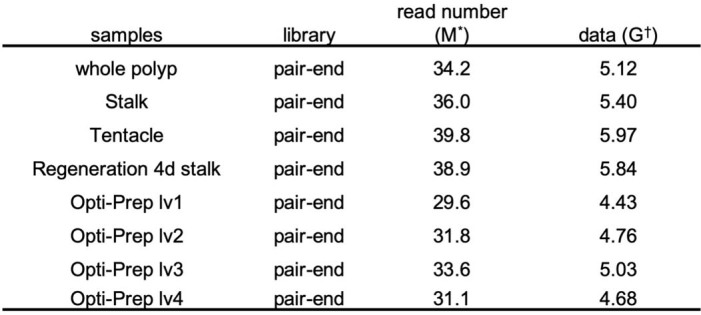
Transcriptomes for gene annotation

A summary of all the transcriptome data used for gene annotation. RNA isolated from different samples as indicated were used for Illumina sequencing to cover as many expressed genes as possible. Opti-Prep, density-based separation of dissociated *Xenia* cells into four different layers (Methods). lv1, lv2, lv3, and lv4 indicate layer 1, layer 2, layer 3 and layer 4 cells, respectively (used to make the RNA-seq libraries). M*, million; G^†^, gigabase.

**Extended Data Table 3:**
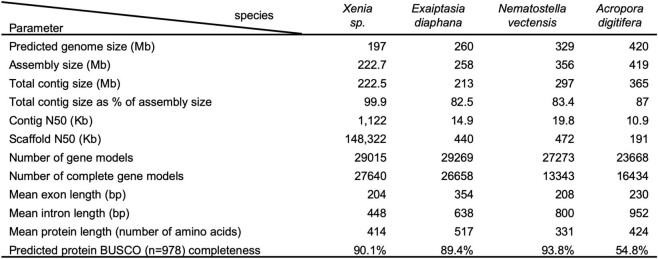
Comparisons of *Xenia* sp. genome assembly with the assembled genomes of the indicated and published cnidarians

The number of gene models indicates the predicted gene model number, whereas the number of complete gene models represents the number of genes with clearly predicted in-frame start and stop codons. Benchmarking Universal Single-Copy Orthologs (BUSCO) completeness was assessed by conserved gene models in metazoans using BUSCO3. The *Exaiptasia diaphana* gene model v.1.0 was downloaded from http://aiptasia.reefgenomics.org/download/aiptasia_genome.proteins.fa.gz. The *Acropora digitifera* gene model v.0.9 was downloaded from https://marinegenomics.oist.jp/coral/download/adi_aug101220_pasa_gene.fa.gz. The *Nematostella vectensis* gene model was downloaded from ftp://ftp.uniprot.org/pub/databases/uniprot/current_release/knowledgebase/reference_proteomes/Eukaryota/UP000001593_45351.fasta.gz.

## Data Availability

We have uploaded all raw genomic, bulk RNA-seq and scRNA-seq data to NCBI (BioProject PRJNA548325). The genome files are available at http://cmo.carnegiescience.edu/data; we have also made the genome data interactive using UCSC genome browser, http://genome.ucsc.edu/cgi-bin/hgTracks?hubUrl=http://cmo.carnegiescience.edu/gb/hub.txt&genome=xenSp1. We allow anyone interested to explore the predicted proteomes of *Xenia* and 14 other cnidarian using our blast server: http://c-moor.carnegiescience.edu:4567. All scRNA-seq analyses and results are available at GitHub: https://github.com/ciwemb/endosymbiosis. Select intermediate RDS objects are available at: http://cmo.carnegiescience.edu/data. We have worked to prototype a web portal to organize all the above links. This work-in-progress has a goal of making research findings, experimental protocols and computational data available to the scientific community. As the portal involves information beyond this study, we are still working with colleagues to best design it so that it will be easy to use and informative. The portal can be accessed at: http://cmo.carnegiescience.edu. Source data Source Data are provided with this paper.

## Code availability

R Markdown codes are available at https://github.com/ciwemb/endosymbiosis. For convenience, processed data and code can be downloaded with the following Unix commands: git clone https://github.com/ciwemb/endosymbiosis; wget -r -np -nH --reject = “index.html*” http://cmo.carnegiescience.edu/endosymbiosis.
