# Supplementary material for: Lineage dynamics of the endosymbiotic cell type in the soft coral Xenia
Source: Nature. 2020 Jun 17;582(7813):534–8. doi: 10.1038/s41586-020-2385-7 (PMC7332420; doi:10.1038/s41586-020-2385-7)
Supplement: Supplementary file 1 — Reporting Summary [file 41586_2020_2385_MOESM1_ESM.pdf]

nature research

Corresponding author(s): Minjie Hu, Chen-Ming Fan, Yixian Zheng

Last updated by author(s): Apr 12, 2020

Reporting Summary

Nature Research wishes to improve the reproducibility of the work that we publish. This form provides structure for consistency and transparency in reporting. For further information on Nature Research policies, see Authors & Referees and the Editorial Policy Checklist.

Statistics

For all statistical analyses, confirm that the following items are present in the figure legend, table legend, main text, or Methods section.

n/a	Confirmed	
☐	☒	The exact sample size (*n*) for each experimental group/condition, given as a discrete number and unit of measurement
☐	☒	A statement on whether measurements were taken from distinct samples or whether the same sample was measured repeatedly
☐	☒	The statistical test(s) used AND whether they are one- or two-sided<br>*Only common tests should be described solely by name; describe more complex techniques in the Methods section.*
☒	☐	A description of all covariates tested
☐	☒	A description of any assumptions or corrections, such as tests of normality and adjustment for multiple comparisons
☐	☒	A full description of the statistical parameters including central tendency (e.g. means) or other basic estimates (e.g. regression coefficient) AND variation (e.g. standard deviation) or associated estimates of uncertainty (e.g. confidence intervals)
☐	☒	For null hypothesis testing, the test statistic (e.g. *F*, *t*, *r*) with confidence intervals, effect sizes, degrees of freedom and *P* value noted<br>*Give P values as exact values whenever suitable.*
☒	☐	For Bayesian analysis, information on the choice of priors and Markov chain Monte Carlo settings
☒	☐	For hierarchical and complex designs, identification of the appropriate level for tests and full reporting of outcomes
☐	☒	Estimates of effect sizes (e.g. Cohen's *d*, Pearson's *r*), indicating how they were calculated

Our web collection on statistics for biologists contains articles on many of the points above.

Software and code

Policy information about availability of computer code

Data collection	MinKNOW (v1.7.3) , Albacore (v2.3.3), bcl2fastaq (v2.20.0)
Data analysis	Canu (v1.7), Nanopolish (v0.9.2), HaploMerger2, Juicer (v1.5), Funannotate (v1.3.3),Trinity (v2.6.6), PASApipeline (v2.3.2), AUGUSTUS (v3.2.3), GeneMark-ES Suite (v4.32), eggnog-mapper (v1.3), OrthoFinder (v2.2.7), Diamond (v0.9.21), MAFFT (v7.407), FastTree (v2.1.10), Cell Ranger (v3.1.0), Seurat (v3.0.2), STAR (v2.5.3a), RSEM (v1.3.0), Monocle (v2.10.1), velocyto.R (v0.6), R(v3.6)

For manuscripts utilizing custom algorithms or software that are central to the research but not yet described in published literature, software must be made available to editors/reviewers. We strongly encourage code deposition in a community repository (e.g. GitHub). See the Nature Research guidelines for submitting code & software for further information.

Data

Policy information about availability of data

All manuscripts must include a data availability statement. This statement should provide the following information, where applicable:

- Accession codes, unique identifiers, or web links for publicly available datasets
- A list of figures that have associated raw data
- A description of any restrictions on data availability

Raw sequence data for this study is available in NCBI BioProject under accession PRJNA548325. Assembled genome and gene annotation are available at http://cmo.carnegiescience.edu/data. The scRNA analysis code is available at https://github.com/ciwemb/endosymbiosis

Field-specific reporting

Please select the one below that is the best fit for your research. If you are not sure, read the appropriate sections before making your selection.

☒ Life sciences ☐ Behavioural & social sciences ☐ Ecological, evolutionary & environmental sciences

For a reference copy of the document with all sections, see nature.com/documents/nr-reporting-summary-flat.pdf

Life sciences study design

All studies must disclose on these points even when the disclosure is negative.

Sample size	No statistical methods were used to predetermine sample size. We followed standards in the biology field.
Data exclusions	For single cell RNA-seq analysis, based on pre-established criteria for single-cells, in order to remove empty droplet, or droplet with potential dead cells or potential doublets, cells with UMI numbers less than 400 or mitochondria gene expression >0.2% were filtered out. To further remove outliers, we calculated the UMI number distribution detected per cell and removed cells in the top 1% quartile.
Replication	Each experiment was replicated with multiple independent animals. To draw a conclusion, at least two independent experiments were carried. All replicates were successful.
Randomization	Xenia colonies or polyps were randomly chosen from the aquarium tank
Blinding	Quantification of LePin signal was blinded by de-identifying samples.

Reporting for specific materials, systems and methods

We require information from authors about some types of materials, experimental systems and methods used in many studies. Here, indicate whether each material, system or method listed is relevant to your study. If you are not sure if a list item applies to your research, read the appropriate section before selecting a response.

Materials & experimental systems

n/a	Involved in the study	
☐	☒	Antibodies
☒	☐	Eukaryotic cell lines
☒	☐	Palaeontology
☐	☒	Animals and other organisms
☒	☐	Human research participants
☒	☐	Clinical data

Methods

n/a	Involved in the study	
☒	☐	ChIP-seq
☐	☒	Flow cytometry
☒	☐	MRI-based neuroimaging

Antibodies

Antibodies used	mouse anti-BrdU antibody, from ZYMED. The catlog number is 18-0103, ZBU30 clone, Lot Number 00460071R. The dilution is 1:200.
Validation	The BrdU antibody was validated by a lot of studies listed in the manufactory's website: https://www.thermofisher.com/antibody/product/BrdU-Antibody-clone-ZBU30-Monoclonal/03-3900. It has been applied in IF, IHC, FACS in Chemical, Chicken, Mouse, Rabbit and Rat. We validated it by the lack of staining when BrdU was not added into the sample.

Animals and other organisms

Policy information about studies involving animals; ARRIVE guidelines recommended for reporting animal research

Laboratory animals	Xenia sp. was cultured in laboratory aquarium tank. We can not yet tell their age and sex.
Wild animals	The Xenia sp. used in this study was originally from the wild, but we obtained it from an aquarium shop in Baltimore.
Field-collected samples	The study didn't involve samples collected from field
Ethics oversight	The study of Xenia or some other cnidaria does not yet have ethical oversight.

Note that full information on the approval of the study protocol must also be provided in the manuscript.

Flow Cytometry

Plots

Confirm that:

☒ The axis labels state the marker and fluorochrome used (e.g. CD4-FITC).

☒ The axis scales are clearly visible. Include numbers along axes only for bottom left plot of group (a 'group' is an analysis of identical markers).

☒ All plots are contour plots with outliers or pseudocolor plots.

☒ A numerical value for number of cells or percentage (with statistics) is provided.

Methodology

Sample preparation	Xenia polyps were dissociated into single cell suspension with the same method for single cell RNA-seq. More details are provided in the method.
Instrument	BD FACSAria™ III
Software	BD FACSDiva Software v6.1.3
Cell population abundance	All Xenia cells were divided into two population, algea-containing and algea-free, based on Cy5.5 signal. The two population have distinct Cy5.5 signal and are easy to separate. There's almost no contamination as confirmed by microscopy inspection on the sorted population. The EdU positive algea-containing population is a small population and the percentage is plotted in Extended fig 7i
Gating strategy	Detailed gating strategy is described in the method.

☒ Tick this box to confirm that a figure exemplifying the gating strategy is provided in the Supplementary Information.
